# Evaluation of distinct and similar pathological features between diabetes and Alzheimer’s disease

**DOI:** 10.17179/excli2026-9429

**Published:** 2026-07-17

**Authors:** Joshua Reed, Stephen C. Bain, Venkateswarlu Kanamarlapudi

**Affiliations:** 1 Institute of Life Science, Medical School, Swansea University, Swansea SA2 8PP, UK

**Keywords:** type 2 diabetes, type 1 diabetes, Alzheimer's disease, islet beta-cell, islet alpha-cell, insulin resistance

## Abstract

Type 1 diabetes (T1D), type 2 diabetes (T2D) and Alzheimer's disease (AD), which are heterogeneous multifactorial diseases, have become major global healthcare burdens and significant concerns for the future. Historically, the pathogeneses of these diseases were regarded as being largely distinct, but recent research suggests that this view is now outdated, with a lack of insulin and/or insulin resistance playing an important role in both forms of diabetes and AD. The current state of knowledge on the epidemiology, aetiology, and pathogenesis of T1D, T2D, and AD is summarised in this review. The evidence demonstrating overlapping pathological features between these diseases is also discussed here. Studies have demonstrated that islet autoimmunity, dysfunctional glucagon secretion, insulin resistance and islet amyloidosis manifest in both T1D and T2D. AD has been suggested to be type 3 diabetes (T3D), due to the associated abnormal brain insulin resistance, which is speculated to drive the progressive pathogenesis. Additionally, T1D and T2D are risk factors for AD and vice versa, and most AD patients also have diabetes or glucose intolerance, indicating bidirectional relationships. Thus, the association between AD and diabetes is already well established. Further understanding the connection between these diseases using recent scientific evidence may allow for the development of more efficient therapeutic strategies, which is highly desirable, given the prevalence of post-diagnosis complications. Evidence suggests that GLP-1R agonists can alleviate the phenotypes of all of these diseases, and these agonists could be used in the future to simultaneously treat them in subjects or prevent/delay their manifestation in at-risk individuals.

See also the graphical abstract(Fig. 1).

## Introduction

Emerging evidence suggests that the major forms of diabetes mellitus, type 1 and 2 diabetes (T1D and T2D), and Alzheimer's disease (AD) share overlapping pathological mechanisms that contribute to the disease phenotypes (Beery et al., 2019[33]; Brooks-Worrell et al., 2022[48]; Donga et al., 2013[99]; Guo et al., 2021[133]; Nguyen et al., 2020[219]; Peng et al., 2024[240]; Reed et al., 2021[262]). This implies that these diseases are not as distinct as currently thought. T1D is an autoimmune disorder characterised by potent immune destruction of islet beta-cells and subsequent hypoinsulinaemia and hyperglycaemia, which results in subjects requiring lifelong insulin treatment to survive (Addissouky et al., 2024[4]; Giwa et al., 2020[130]; Richardson et al., 2011[267]). T2D, however, is characterised as a metabolic disorder largely manifested by impaired islet beta-cell insulin secretion and systemic insulin resistance, due to chronic excessive nutrient intake in overweight individuals (Reed et al., 2021[262]). T1D usually develops more rapidly than T2D and requires immediate lifelong insulin therapy to survive, due to the greater insulin deficiency induced by the islet beta-cell autoimmunity (Atkinson et al., 2014[22]; Reed et al., 2021[262]). Evidence has emerged that T1D and T2D both share pathological features that were previously thought to be unique to each form of diabetes, which challenges the widely accepted notion that these diseases are distinct (Beery et al., 2019[33]; Brooks-Worrell et al., 2022[48]; Donga et al., 2013[99]; Guo et al., 2021[133]; Reed et al., 2021[262]). AD is the most common form of dementia and has been suggested by some to be T3D, given the evidence demonstrating that the disease-associated pathology is induced by the manifestation of chronic insulin resistance that selectively manifests in the brains of subjects (de la Monte and Wands, 2008[90]; Kamatham et al., 2024[171]; Nguyen et al., 2020[219]; Peng et al., 2024[240]). AD and diabetes pathology are known to be strongly associated, as the majority of AD patients have T2D or impaired glucose tolerance (Barbagallo and Dominguez, 2014[28]). Interestingly, AD subjects who do not have T2D or impaired glucose tolerance have also been shown to be at increased risk of developing these conditions post-AD diagnosis (Janson et al., 2004[163]), which is not well-known or studied to date. This review summarises the current knowledge of the epidemiology, aetiology and pathogenesis of T1D, T2D and AD, and attempts to highlight the evidence demonstrating that these diseases are not as distinct as previously thought, given some of the pathological features shared between them and how diabetes and AD increase the risk of each other manifesting in subjects, indicating bidirectional relationships and a continuum of metabolic dysfunction. Further understanding the similar pathology between these diseases may allow for the development of more efficient therapeutic strategies that alleviate the disease phenotypes to a greater extent than current treatments.

## Diabetes Mellitus

Diabetes mellitus (DM) encompasses a group of disorders largely characterised by impaired carbohydrate metabolism, where glucose is underutilised as an energy source and is overproduced due to inappropriate gluconeogenesis and glycogenolysis, resulting in hyperglycaemia (American Diabetes Association Professional Practice Committee, 2025[12]). DM has become a global pandemic in recent decades, due to the rise in the prevalence and incidence globally of T1D and T2D (Guariguata et al., 2014[132]; Reed, 2021 et al.[262]; Saeedi et al., 2019[270]). DM is therefore a major global healthcare burden, with an estimated 425 million people affected globally in 2017 (Saeedi et al., 2019[270]), and the number of patients is expected to rise to 590 million by 2035 (Reed, 2021 et al.[262]; Saeedi et al., 2019[270]). The majority of people with diabetes, approximately 90 %, are diagnosed with T2D, and the remaining approximately 10 % are diagnosed with T1D or other forms (Reed et al., 2024[263]). Despite available treatments, complications of DM remain prevalent globally (Reed et al., 2021[262]). Diabetes is a leading cause of cardiovascular disease (CVD), blindness, leg amputation and end-stage renal disease (Brownlee, 2001[52]). An estimated 75 % of deaths in people with diabetes are due to cardiovascular complications (Ali et al., 2010[9]; Leal et al., 2009[181]). Individuals with diabetes also have an increased risk of developing dementia (Cholerton et al., 2016[68]; Li et al., 2025[184]). Currently, it is widely accepted that both forms of diabetes have distinct aetiologies, with T1D being an autoimmune disorder and T2D being a metabolic disorder (Lu et al., 2024[197]; Ojo et al., 2023[227]; Sanyaolu et al., 2023[276]; Zajec and Trebušak Podkrajšek, 2022[341]).

## Type 2 Diabetes

T2D has become a global pandemic in recent decades and it is a highly heterogeneous disease, as insulin secretion/levels, systemic insulin resistance, treatment requirements, age of onset and prognosis vary between subjects (Reed et al., 2021[262]). T2D manifests stereotypically in overweight individuals, as the chronic strain of the excessive nutrient intake promotes systemic insulin resistance and eventual impaired insulin secretion by islet beta-cells (Cerf, 2013[62]; Reed et al., 2021[262]; Singh et al., 2025[288]; Taylor, 2024[304]). When islet beta-cell function declines to such an extent that hyperglycaemia manifests, this results in the clinical manifestation of the disease (Boland et al., 2017[40]; Cerf, 2013[62]; Reed et al., 2021[262]). T2D pathogenesis is complex, involving currently mechanistically unclear interactions between diet/lifestyle, genetics and environment that initiate the condition (Reed et al., 2021[262]). Research over recent decades has revealed that T2D is a highly complex multifactorial disease. Whilst significant progress has been made in understanding all its aspects, such as its aetiology and pathogenesis, and developing treatment strategies, much remains to be elucidated, and more effective therapeutic options are needed (Lu et al., 2024[197]; Reed et al., 2021[262], 2024[263]; Singh et al., 2025[288]; Taylor, 2024[304]).

### Epidemiology

**Figure 2:**
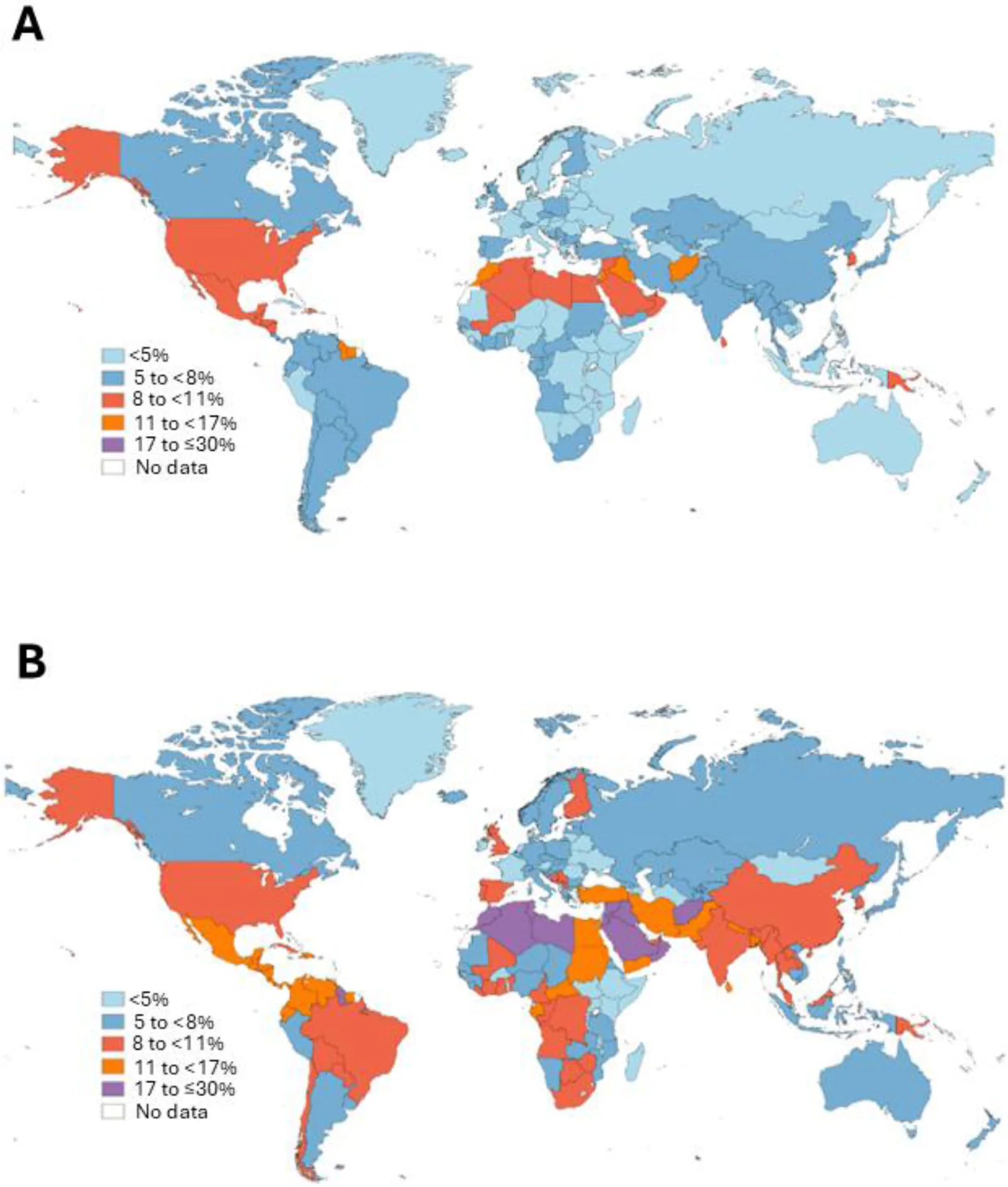
Global age-standardised prevalence rates of T2D in 2021 (A) and 2050 (B). This figure was generated with the data taken from (Ong et al., 2023).

Both the prevalence and incidence of T2D have increased greatly over the last few decades worldwide (Guariguata et al., 2014[132]; Ozougwu et al., 2013[234]; Reed et al., 2021[262]). T2D used to be a rare disease, with < 1 % of the population in the USA and China being affected in 1958 and 1980, respectively (Ginter and Simko, 2012[129]; Reed et al., 2021[262]; Weng et al., 2016[327]). Additionally, in some nations, it has become increasingly common for children and adolescents to develop T2D (Onge et al., 2015[231]; Reinehr, 2013[266]), with an unprecedented ~80 % of new cases in Japan not being adults (D'Adamo and Caprio, 2011[84]). Hence, T2D has shifted from being a historically rare adult disease to becoming a major global medical bane that can develop before adulthood (Reed et al., 2021[262]). The major increase in T2D prevalence and incidence was first observed in developed countries like the USA, but this increase has also been mirrored in developing countries (Ginter and Simko, 2012[129]; Guariguata et al., 2014[132]). In the future, the increased global rise in T2D cases is estimated to be largely due to a rise in prevalence, with its incidence largely stabilising in some nations (Reed et al., 2021[262]). Figure 2(Fig. 2) (References in Figure 2: Ong et al., 2023[230]) shows how the age-standardised prevalence rates of T2D varied between countries in 2021 and projected data for 2050.

### Aetiology

Given that an estimated 90 % of people with diabetes are overweight, T2D pathogenesis is induced by excessive caloric diets with sedentary lifestyles, although it is still not clear how mechanistically (Reed et al., 2021[262], 2024[263]). The strong positive correlation between T2D risk and increasing BMI has led to the widely accepted notion that T2D is induced by poor diet and lifestyle (Reed et al., 2021[262]). However, it remains unclear why individuals of normal weight or even underweight subjects develop this condition (Mathews and Mathews, 2012[202]; Reed et al., 2021[262]; Zhou et al., 2016[351]). Studies have found that in Asian populations, many subjects are not overweight, with one study finding that > 50 % of the individuals were not overweight (BMI under 25 Kg/m^2^) in Japan (George et al., 2015[126]; Ma and Chan, 2013[199]; Mathews and Mathews, 2012[202]). Additionally, obese and overweight subjects have been reported to have had a lower mortality rate than normal-weight subjects due to T2D-associated cardiovascular pathology, termed “the obesity paradox” (Costanzo et al., 2015[77]; Hainer and Aldhoon-Hainerová, 2013[135]). Interestingly, studies have also shown that glycaemic control is worse in non-overweight T2D people (Coleman et al., 2014[73]; George et al., 2015[126]). Therefore, the existence of non-overweight T2D individuals with worse prognosis challenges the notion that T2D pathology is solely induced by lifestyles/diets that promote weight gain/obesity. The current consensus is that the islet beta-cell dysfunction is greater in non-overweight subjects and systemic insulin resistance is less pronounced (Coleman et al., 2014[73]; George et al., 2015[126]; Reed et al., 2021[262]), and as insulin resistance is induced by excessive nutrient storage systemically due to excessive diets/sedentary lifestyles, this seems logical. However, it is unclear/paradoxical why islet beta-cell dysfunction is present to a greater extent in non-overweight people, as islet beta-cell pathology in T2D is accepted to be promoted by excessive ingestion of nutrients resulting in weight gain (Reed et al., 2021[262]). Therefore, assuming this notion applies to all T2D individuals, increased islet beta-cell dysfunction should be observed in overweight subjects in comparison, which has not been reported by several studies (Coleman et al., 2014[73]; George et al., 2015[126]; Reed et al., 2021[262]).

Given that > 72 % of the global overweight population, estimated to have exceeded 2.8 billion in 2022 (WHO, 2025[331] ), have not been diagnosed with T2D (WHO, 2024[330]) and many overweight individuals do not even develop insulin resistance (Reed et al., 2021[262]), with one study finding that 66 % of individuals with a BMI ≥ 30-35 did not exhibit insulin resistance (Ferrannini et al., 1997[113]), demonstrates that diabetic pathology is not exclusively induced by excessive diets and poor lifestyles. Genetics has been identified as increasing susceptibility to T2D. The lifetime risk of developing T2D with one affected parent is ~40 % and ~70 % if both parents are affected (Ahlqvist et al., 2011[5]; Chen et al., 2011[64]; Prasad and Groop, 2015[252]). It is currently unclear if the increased susceptibility of relatives of T2D to develop this disease could be induced by them having similar diets and lifestyles, as well as genetics (Chen et al., 2011[64]; Poulsen et al., 2009[251]). One study demonstrated the association of genetics with T2D seemingly independently of diet/lifestyle, as disease prevalence and incidence were higher in the twin population compared to the singleton population; both of these populations had similar BMI scores (Poulsen et al., 2009[251]). Genome-wide association studies (GWAS) have found > 700 variants that confer small-to-modest susceptibility to T2D, as odds ratios reported for any individual loci do not exceed 1.6 to date, which implies there are currently untested variants that confer increased susceptibility 24256 (Imamura and Maeda, 2024[154]; Prasad and Groop, 2015[252]). In addition to unhealthy lifestyles and genetics, other risk factors have also been identified for T2D, such as alcohol consumption, smoking and psychological stress (Merabet et al., 2022[209]; Mozaffarian et al., 2009[216]; Reed et al., 2021[262]).

### Pathogenesis

During weight gain, individuals develop systemic insulin resistance, which then initially induces islet beta-cells to increase their insulin content and secretion to compensate and maintain euglycemia (Fonseca, 2009[116]; Linnemann et al., 2014[187]; Mehran et al., 2012[208]). Over time, usually over a period of several years or more, the islet-beta cells are seemingly unable to cope with the increased burden and individuals insidiously progress to prediabetes, where blood glucose levels rise due to declining islet beta-cell function, but not to such an extent that this warrants T2D diagnosis (Bansal, 2015[27]; Tabák et al., 2012[300]). Although lifestyle interventions, such as weight loss and exercise, can delay or prevent progression to T2D or even reverse prediabetes, an estimated 70 % of individuals with persistent prediabetes eventually progress to T2D (Bansal, 2015[27]; Tabák et al., 2012[300]; Tuso, 2014[314]). T2D manifests clinically when symptoms appear due to hyperglycaemia (Olokoba et al., 2012[228]; Reed et al., 2021[262]). Individual's blood glucose levels rising to > 7 mmol during fasting and > 11 mmol two hours after 75g glucose ingestion warrant T2D diagnosis; subjects typically then require lifelong treatments to try to achieve euglycemia (Bansal, 2015[27]; Davidson et al., 2000[86]). In T2D, people's fasting insulin levels in the circulation are estimated to decrease by ~50 % and postprandial levels in subjects are estimated to be between 25-40 % of what is observed in diabetes-free weight-matched controls (Woerle et al., 2012[334]). Typically, T2D is a progressive disease, and ~75 % of subjects progress to requiring more than one pharmacological agent to alleviate hyperglycaemia within ten years of diagnosis (Reed et al., 2021[262]; Taylor, 2013[303]).

Although the widely accepted notion is that the T2D disease phenotype is induced by a heterogeneous combination of systemic insulin resistance and impaired insulin secretion, emerging evidence suggests that multiple other pathological mechanisms contribute to the disease pathogenesis/phenotype (Reed et al., 2021[262]; Sanches et al., 2023[274]). Islet beta-cell apoptosis is known to be increased in T2D individuals (Butler et al., 2003[54]; Khin et al., 2023[174]; Reed et al., 2021[262]), with greater levels of apoptosis observed in those with a BMI < 25 Kg/m^2^ (Butler et al., 2003[54]), which likely additionally contributes to the insulin deficit in T2D. Islet amyloid polypeptide (IAPP) plaques in pancreatic islets, formed by misfolding of IAPP, have been found in the majority, > 90 %, of T2D individuals (Gupta and Leahy, 2014[134]; Smith et al., 2022[289]). Currently, it is thought that extracellular plaques of IAPP do not induce islet beta-cell pathology, but rather intracellular oligomers of IAPP produce cytotoxic effects, which contribute to the insulin deficit in T2D (Alrouji et al., 2023[10]; Gupta and Leahy, 2014[134]; Matveyenko and Butler, 2006[203]). Cytotoxic effects of intracellular IAPP reported by *in vitro* studies include increased generation of reactive oxygen species (ROS) and induction of endoplasmic reticulum (ER) stress, which likely contribute to both impaired insulin secretion and increased apoptosis in people with T2D, assuming the situation is similar in *in vivo *settings (Alrouji et al., 2023[10]; Casas et al., 2007[60]; Pilkington et al., 2016[247]). The precise mechanisms by which these plaques promote T2D in individuals are currently unclear (Smith et al., 2022[289]). Autophagic processes usually protect islet beta-cells from intracellular accumulation of IAPP oligomers, so it seems that these processes are impaired in T2D (Gupta and Leahy, 2014[134]; Shigihara et al., 2014[286]). T2D-free individuals also have IAPP deposits detected in their islets, but with a greatly reduced incidence compared to T2D subjects (Butler et al., 2003[54]; Reed et al., 2021[262]), which implies that the presence of these plaques alone does not induce disease.

Islet alpha-cell dysfunction, where glucagon is undesirably released into the circulation in T2D during the postprandial period, further worsens the hyperglycaemia (Moon and Won, 2015[214]; Sherwin et al., 1976[284]). It has been shown that the postprandial hyperglucagonemia can contribute to up to 50 % of the hyperglycaemia in individuals with T2D after glucose ingestion. T2D is, therefore, now considered to be a bihormonal disorder (Dinneen et al., 1995[97]; Moon and Won, 2015[214]; Reaven et al., 1987[260]; Shah et al., 2000[281]; Sherwin et al., 1976[284]; Shigeno et al., 2021[285]). Islet beta-cells are unresponsive to the incretin action of gastric inhibitory peptide (GIP), even at supraphysiological concentrations in T2D (Højberg et al., 2008[149]; Reed et al., 2021[262]). Hence, the impaired incretin effect also contributes to insufficient postprandial insulin secretion in T2D. Chronic islet inflammation is also associated with T2D, which is thought to contribute to islet beta-cell dysfunction, evidenced by alleviation of the disease phenotype by anti-inflammatory therapies in several studies (Cuenco and Dalmas, 2022[79]; Donath et al., 2009[98]; Eguchi and Nagai, 2017[108]). It has been demonstrated in both rodent (Ehses et al., 2009[109]; Sauter et al., 2008[277]) and human (Larsen Claus et al., 2007[180]; Rissanen et al., 2012[268]) studies that interfering with the IL-1 pathway reduced hyperglycaemia and improved islet beta-cell function. Additionally, extrapancreatic pathology, besides systemic insulin resistance, contributes to the disease phenotype; research suggests that some of these pathologies manifest during prediabetes, implying they are involved in T2D aetiology and pathogenesis (Basu et al., 2013[32]; Phielix and Mensink, 2008[244]; Reed et al., 2021[262]). Impaired fat catabolism and metabolic inflexibility in the skeletal muscle of T2D subjects have been observed, which contribute to the disease phenotype, as well as reduced insulin suppression of hepatic gluconeogenesis and glycogenolysis during prediabetes (Basu et al., 2013[32]; Phielix and Mensink, 2008[244]). The extrapancreatic pathologies, besides systemic insulin resistance, involved in exacerbating the T2D phenotype are reviewed in more detail elsewhere (Reed et al., 2021[262]).

## Type 1 Diabetes

T1D is an autoimmune disorder that is fatal without treatment due to the absolute insulin deficiency (Perilli et al., 2013[241]; Reed et al., 2021[262]). T1D pathogenesis is induced by autoimmune destruction of islet beta-cells mediated by autoreactive T cells and antibodies (Addissouky et al., 2024[4]; Giwa et al., 2020[130]; Zajec and Trebušak Podkrajšek, 2022[341]). The T1D-associated autoimmunity pathogenesis is complex, involving currently mechanistically unclear interactions between genetics and environment that initiate loss of immunological tolerance (Addissouky et al., 2024[4]; Giwa et al., 2020[130]; Michels et al., 2022[210]; Zajec and Trebušak Podkrajšek, 2022[341]). After the initiation of islet beta-cell autoimmunity, function and mass of these cells decline progressively, and the time taken for symptoms and hyperglycaemia to manifest can vary greatly between individuals for currently unknown reasons (Addissouky et al., 2024[4]; Gillespie and Long, 2019[128]; Insel et al., 2015[155]). T1D progression is currently split into three distinct stages, with an asymptomatic stage 1 diagnosis requiring individuals to be positive for 2 or more islet autoantibodies and having normal glucose levels, and individuals remain asymptomatic in stage 2 but have elevated glucose levels (dysglycaemia) during an oral glucose tolerance test (Aamodt and Powers, 2025[1]; O'Donovan et al., 2025[224]). Diabetes diagnosis (stage 3 T1D) occurs upon manifestation of symptoms and hyperglycaemia (Aamodt and Powers, 2025[1]; Addissouky et al., 2024[4]; Bartoli et al., 2011[31]). Individuals with T1D then require lifelong insulin treatment to survive unless they undergo a pancreas transplantation, which is a major operation also limited by the low number of donor organs from deceased individuals (Addissouky et al., 2024[4]; Hazime et al., 2025[142]). Research has elucidated that T1D is a complex multifactorial disease, and although much progress has been made in understanding its aetiology and pathogenesis, more effective therapeutic options are highly desirable (Addissouky et al., 2024[4]; Giwa et al., 2020[130]; Zajec and Trebušak Podkrajšek, 2022[341]).

### T1D epidemiology

**Figure 3:**
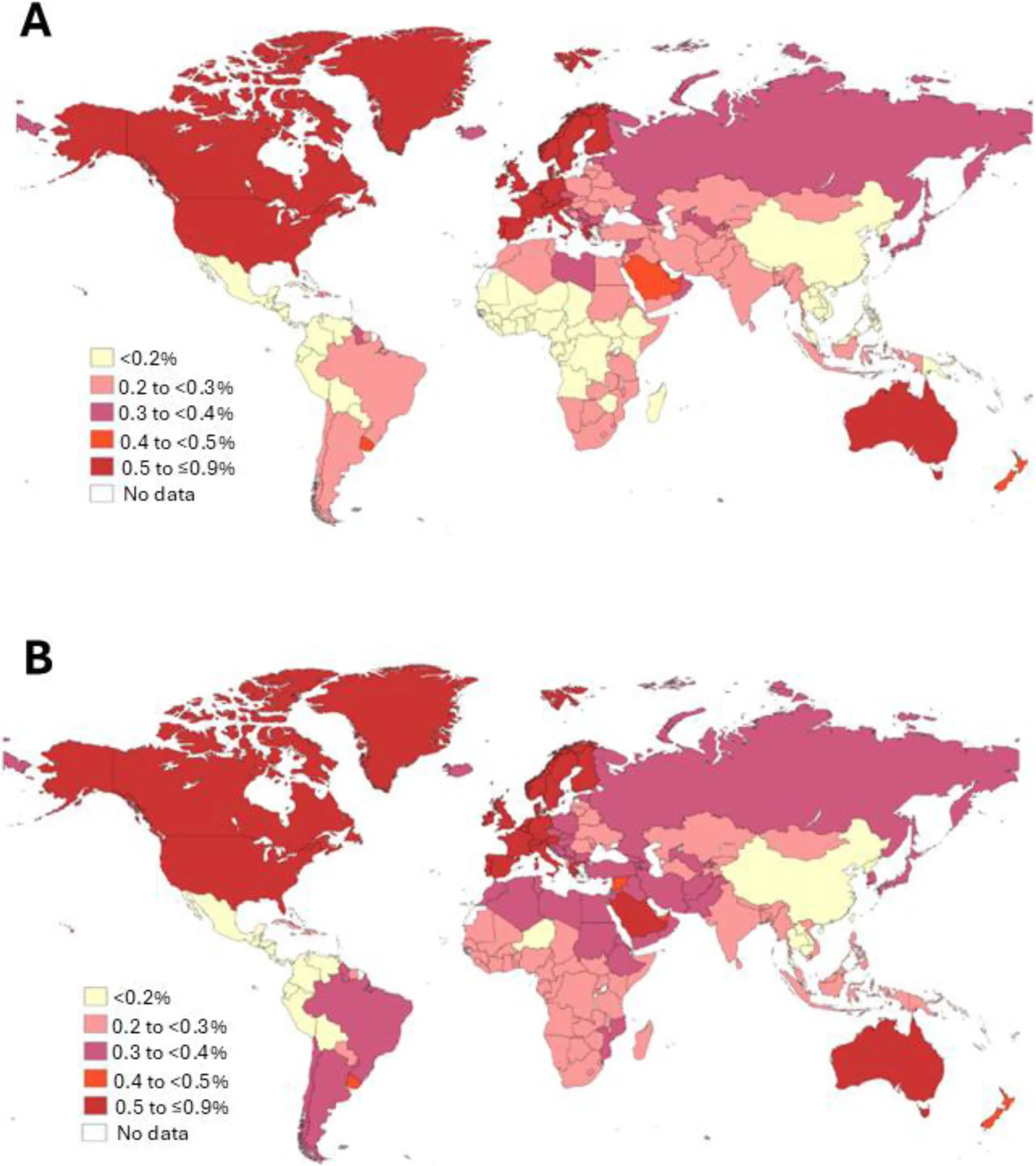
Global age-standardised prevalence rates of T1D in 2021 (A) and 2050 (B). This figure was generated with data taken from (Ong et al., 2023).

Overall, the incidence and prevalence of T1D increase each year globally, and there were an estimated 9.2 million patients worldwide in 2024 (Bell and Lain, 2025[34]). Historically, the majority of T1D subjects were diagnosed during childhood and adolescence (Haller et al., 2005[136]; Karvonen et al., 2000[172]; Maahs et al., 2010[200]), but recent studies have found that adult-onset T1D has become increasingly common, even more common than childhood-onset (Leslie et al., 2021[183]). Interestingly, the incidence of T1D has increased greatly in the modern world globally, and in Finland, the incidence has increased > 4-fold since the early 1950s (Virtanen and Knip, 2003[323]). Between 1990 and 1994, the incidence of T1D was determined in children ≤ 14 years in 50 countries (Karvonen et al., 2000[172]; Maahs et al., 2010[200]); T1D incidence was much higher in Caucasian populations, with incidence rates of > 36/100,000 per year in Finland and Sardinia. In contrast, in China and Venezuela, T1D incidence was drastically lower, an estimated 0.1/100,000 per year. Therefore, although T1D has a global clinical presence, the associated healthcare burden of this disease varies greatly between nations and ethnicities (Ogle et al., 2025[226]). Surprisingly, subsequent studies have reported enormous increases in T1D incidence in low-risk countries such as China (Liu et al., 2021[188] ; Ogle et al., 2025[226]; Weng et al., 2018[328]). One study reported T1D incidence between 2010-2013 in China was 1.93/100,000 for 0-14 years, which is an approximately 19-fold increase from what was previously found (Weng et al., 2018[328]). Another study found that T1D incidence in China was 4.21/100,000 for the same age group in 2017 (Liu et al., 2021[188]), and overall T1D incidence in China went from 2.72 in 2007 to 3.6/100,000 in 2017. A recent study estimated that T1D incidence for subjects under 15 years in 2025 was 4.8 and 4.9/100,000 for China and Venezuela, respectively (Ogle et al., 2025[226]). These incidence rates are nearly 50-fold higher than those reported for these countries during 1990-1994. The drastic increase in T1D incidence in these low-risk countries over the last few decades warrants thorough investigation. It has been suggested that the earlier studies may have underestimated T1D incidence in China (Liu et al., 2021[188]), which could account for at least part of the reported increase. Figure 3(Fig. 3) (References in Figure 3: Ong et al., 2023[230]) shows how the age-standardised prevalence rates of T1D varied between countries in 2021 and projected data for 2050 are also shown.

### Autoantibodies in T1D

 Autoantibodies against insulin (IAA), glutamic acid decarboxylase (GADA), islet antigen 2 (IA-2A) and zinc transporter 8 autoantibodies (ZnT8A) are biomarkers of islet beta-cell autoimmunity (Hazime et al., 2025[142]; Jia and Yu, 2024[168]). Islet beta-cell autoimmunity is detected clinically by the presence of one or more of these four autoantibodies, and individuals can be consistently positive for these antibodies for months to years before stage 3 T1D manifests (Addissouky et al., 2024[4]; Bosi et al., 2017[44]; Jacobsen et al., 2020[159]). Individuals positive for multiple autoantibodies usually progress to the clinical phase of T1D more rapidly (Bonifacio and Ziegler, 2025[42]; Jamjoom et al., 2024[161]; Ziegler et al., 2013[353]). Screening for all four of these antibodies allows for a > 94 % detection rate of autoimmunity in recent-onset diabetes cases (Lampasona et al., 2010[179]; Long et al., 2012[195]). However, a subset of T1D subjects do not display seropositivity for any of these autoantibodies (Patel et al., 2021[238]). Interestingly, some individuals, known as slow progressors, remain T1D-free after > 10 years of being consistently positive for 2 or more autoantibodies (Achenbach et al., 2013[3]; Gillespie and Long, 2019[128]; Long et al., 2018[196]). Therefore, whilst detection of these autoantibodies demonstrates the presence of type 1 diabetic autoimmunity in subjects, they are not always reliable indicators of how long the preclinical asymptomatic phase lasts before onset of the clinical phase. Additionally, the presence of these antibodies can be transient, and titres can fluctuate in subjects for currently unclear reasons (Achenbach et al., 2005[2]; Phillip et al., 2024[245]; Sørgjerd, 2019[290]). Not all individuals who develop single autoantibodies test positive for them on follow-up screening or progress to develop multiple antibodies or T1D (Vehik et al., 2016[321]). Positivity for the autoantibodies post-diagnosis has been shown to decline with time (Long et al., 2021[193] ; Williams et al., 2022[333]), with one study reporting that < 30 % of subjects had 2 or more autoantibodies > 15 years after diagnosis (Williams et al., 2022[333]). In contrast, individuals can also test positive for one or more of the autoantibodies post-diagnosis (Decochez et al., 2000[91]; Sørgjerd, 2019[290]). Given these observations, it is clear that the humoral autoimmune response has a heterogeneous nature during the preclinical and clinical disease phases for currently unknown reasons. Whilst slow progressors are established in the literature 1 (Achenbach et al., 2013[3]; Gillespie and Long, 2019[128]), it is suggested here that individuals, after testing positive consistently for one or more of the autoantibodies, could be classified as rapid or intermediate progressors if they develop T1D within 5 years or between 5 and 10 years after initial seroconversion, respectively.

### Aetiology

Genetic susceptibility plays a major but not exclusive role in T1D development, with multiple susceptibility genes being identified (Stefan et al., 2014[296]). Concordance rates between identical twins are estimated to be between 30-50 % by several studies (Hyttinen et al., 2003[153]; Stefan et al., 2014[296]; Verge et al., 1995[322]), which indicates the importance of both genetics and environmental factors in conferring T1D susceptibility. The *DR3-DQ2* and *DR4-DQ8* haplotypes of the human leukocyte antigen (HLA) class II genes are widely accepted as the major inherited risk factors for developing T1D (Nygård et al., 2021[223]; Sticht et al., 2021[298]; Zajec and Trebušak Podkrajšek, 2022[341]; Zhao et al., 2021[348]). An estimated 90 % of T1D individuals have one of these high-risk haploty[348]pes and approximately 40 % of subjects have both: in the healthy population, 30 % have one haplotype and 3 % have both (Zajec and Trebušak Podkrajšek, 2022[341]). An estimated > 95 % of individuals who have one of these high-risk haplotypes do not develop T1D (Giwa et al., 2020[130]). Interestingly, these haplotypes predict seroconversion and not age of onset, clinical progression, or C-peptide loss post-diagnosis (Carré et al., 2021[59]; Zajec and Trebušak Podkrajšek, 2022[341]). The Australian Melbourne prediabetes study reported that these high-risk haplotypes were more prevalent in individuals who developed multiple persistent autoantibodies than in individuals who were positive for one or transiently positive for one or more (Colman et al., 2000[74]). To date, more than 40 single-nucleotide polymorphisms (SNPs) outside the HLA region have been associated with T1D susceptibility, although these SNPs only have a small effect on disease risk in comparison to the strong influence of the HLA class II haplotypes (Barrett et al., 2009[30]; Nygård et al., 2021[223]; Törn et al., 2014[312]). Certain HLA class I alleles have also been shown to influence T1D risk (Zajec and Trebušak Podkrajšek, 2022[341]). HLA-A*24 predicts rapid progression to T1D in the presence of islet beta-cell autoimmunity in individuals with the *DR3-DQ2* and/or *DR4-DQ8* haplotypes (Jiang et al., 2021[170]; Long et al., 2013[194]; Mbunwe et al., 2013[204]; Noble and Erlich, 2012[221]; Zajec and Trebušak Podkrajšek, 2022[341]). Interestingly, one study also found that HLA-A*24 confers T1D risk in individuals who have neither high-risk haplotype (Honeyman et al., 1995[151]). HLA-B*39 also confers additional risk in individuals with high- and low-risk haplotypes and is also associated with early age at onset (Mikk et al., 2014[212], 2017[211]; Reijonen et al., 1997[265]). HLA-B*18 has also been shown to contribute to T1D progression in islet autoantibody-positive individuals with the *DR3-DQ2* haplotypes (Mbunwe et al., 2013[204]). Interestingly, the HLA class II haplotype DRB1*15-DQB1*0602 is present in an estimated 20 % of the population but only occurs in around 1 % of people with T1D, and is therefore considered a protective haplotype for T1D (Lambert et al., 2004[178]; Pugliese et al., 1995[253]).

Given the difference in T1D incidence between ethnic groups, it is reasonable to assume that genetic ethnic differences may account for these observations. However, migration studies have demonstrated the importance of geographical/environmental factors, as when populations from low-risk ethnic groups immigrate to Western nations, the risk of their children developing T1D increases greatly (Harron et al., 2011[139]; Hjern et al., 2012[148]; Hussen et al., 2013[152]; Umpierrez and Ali, 2025[315]). Additionally, the strong increase in incidence globally over the last few decades cannot be exclusively attributed to changes in genetic susceptibility, further providing evidence that environmental factors must be playing an increased role in promoting T1D pathogenesis. For unknown reasons, T1D onset is more common during late autumn, winter and early spring (Maahs et al., 2010[200]; Turtinen et al., 2022[313]). One recent study found that the seasonality of T1D manifestation differed between younger and older children, indicating that age and seasonality interact synergistically to promote disease risk (Turtinen et al., 2022[313]). It has also been shown that infants born between November and February are less likely to develop T1D than infants born in April-July (Maahs et al., 2010[200]; Vaiserman et al., 2007[318]). Interestingly, improved hygiene conditions correlate with greater susceptibility to T1D; there is an inversely proportional relationship between T1D incidence and both infectious disease incidence and poor sanitation (Bach, 2021[24]; Bach and Chatenoud, 2012[25]). A rodent study supported this observation, as it was found that non-obese diabetic mice reared in poor sanitary conditions had decreased risk of T1D, and that deliberately infecting them with various pathogens prevented T1D manifestation (Bach and Chatenoud, 2012[25]). However, some studies have found evidence suggesting that viral infection by enteroviruses may promote T1D pathogenesis (Giwa et al., 2020[130]), with studies finding that among children diagnosed with T1D, > 60 % of them had positive coxsackievirus serology (Clements et al., 1995[70]; Friman et al., 1985[120]). Additionally, one study found that coxsackievirus B1 increased the risk of detection of two or more islet autoantibodies in T1D-free children (Laitinen et al., 2014[177]). In contrast, other studies have failed to show any link between enteroviral infections and T1D (Knip and Simell, 2012[176]).

### Pathogenesis

Pancreatic inflammation (insulitis) was initially observed in pancreas samples from patients with recent-onset T1D nearly a century ago (Stansfield and Warren, 1928[294]). In the later part of the 20^th^ century, the cellular composition was elucidated: the vast majority of cells present in the insulitis were lymphocytes, which outnumbered the macrophages by a ratio of 10:1 (Foulis et al., 1991[117]; Gepts, 1965[127]). In recent decades, histological evidence for cytotoxic T cells killing islet beta-cells has emerged and the importance of antigen-specific T cells has been described (Coppieters and von Herrath, 2009[76]; Itoh et al., 1993[158]; Willcox et al., 2009[332]). The current notion is that cytotoxic T cell killing of islet beta-cells in a cell-mediated adaptive immune response, via induction of apoptosis, accounts for the majority of T1D pathogenesis, although the innate immune system is thought to also contribute by encouraging inflammation and T cell migration into the islets (Addissouky et al., 2024[4]; Pirot et al., 2008[248]; Richardson et al., 2011[267]; Yoon and Jun, 2005[339]; Zajec and Trebušak Podkrajšek, 2022[341]). The exact mechanisms of islet beta-cell death in T1D are still debated, but it is thought that following binding of a CD8+ T cell receptor to an autoantigen/HLA complex that it recognises, apoptosis is induced in islet beta-cells by perforin and granzyme activity, as well as Fas binding to Fas ligand, which both result in activation of various caspases and release of cytochrome c from the mitochondria (Long et al., 2012[195]; Richardson et al., 2011[267]; Taylor, 2024[304]; Tomita, 2017[311]; Uno et al., 2007[316]; van Belle et al., 2011[319]). Interestingly, the cellular composition of immune cells associated with the autoimmunity present in the islets can vary between subjects, with some cases having regulatory T cells, B lymphocytes and natural killer cells present (Dotta et al., 2007[100]; Richardson et al., 2011[267]; Willcox et al., 2009[332]). In healthy subjects, HLA class II is not expressed in pancreatic endocrine cells, but low levels of class I are (Richardson et al., 2011[267]). Preceding any inflammation or immune cell influx into the pancreatic islets, HLA class I is upregulated in the majority of islet beta-cells, and subsequently, hyperexpression of HLA class II then follows in a minority of these cells (Bottazzo et al., 1985[45]; Foulis et al., 1991[117]; Richardson et al., 2011[267]; Thomas et al., 1998[307]). It is thought that the increased antigen presentation to T cells then encourages autoimmunity, but it is currently unclear why the increased expression of HLA class I and II occurs (Akhbari et al., 2025[7]; Benkahla et al., 2021[37]; Richardson et al., 2011[267]).

The pathogenesis of T1D then progresses insidiously, as cytotoxic T cells move into the islets and begin attacking islet beta-cells presenting autoantigens, and macrophages are present in small numbers. However, the number of islet beta-cells declines slowly and individuals remain asymptomatic (Addissouky et al., 2024[4]; Richardson et al., 2011[267]). This ongoing autoimmunity can be detected by screening for the aforementioned autoantibodies (Hazime et al., 2025[142]; Jia and Yu, 2024[168]). Over a period of time, which can vary greatly between individuals, progression to late-stage islet beta-cell autoimmunity occurs, which is characterised by a more diffuse inflammatory infiltrate in and around the islets with both increased numbers of cytotoxic T cells and B lymphocytes, and significantly decreased numbers of islet beta-cells (Richardson et al., 2011[267]; Uno et al., 2007[316]). The number of macrophages remains at a similar level in late-stage islet beta-cell autoimmunity and it is thought they contribute to beta-cell demise by releasing pro-inflammatory cytokines such as TNFα and IL-1β (Richardson et al., 2011[267]; Uno et al., 2007[316]; Willcox et al., 2009[332]). It remains unclear whether the T1D autoantibodies mediate any clinically relevant islet beta-cell damage/death during the pre-clinical phase (Addissouky et al., 2024[4]; Richardson et al., 2011[267]; Tomita, 2017[311]; Yoon and Jun, 2005[339]). When islet beta-cell numbers become low during late-stage islet beta-cell autoimmunity (usually 70-80 % destroyed), they can no longer prevent hyperglycaemia, and T1D manifests clinically (stage 3 T1D) (Cnop et al., 2005[72]; Richardson et al., 2011[267]). Interestingly, emerging evidence suggests that whilst the vast majority of islet beta-cells are destroyed in subjects, some persistent low-level beta-cell function is usually present (Odyjewska et al., 2025[225]; Oram et al., 2019[232]). Studies have demonstrated that greater residual islet beta-cell function is associated with lower rates of post-diagnosis complications (Harsunen et al., 2023[140]; Steffes et al., 2003[297]). Currently, it is mechanistically unclear how these cells evade autoimmune destruction or if they formed pre- or post-diagnosis (Oram et al., 2019[232]). Understanding how individuals can maintain a small population of islet beta-cells and their function could yield new therapeutic strategies for T1D.

## Shared Pathological Features between T1D and T2D

Over recent decades, evidence continues to emerge that pathological features that were historically thought to be exclusive to each major form of diabetes are demonstrated in both (Bielka et al., 2024[38]; Bisgaard Bengtsen and Møller, 2021[39]; Brooks-Worrell et al., 2022[48]; Denroche and Verchere, 2018[93]; Guo et al., 2021[133]; Reed et al., 2021[262]). Therefore, the disease phenotypes of T1D and T2D might be induced by similar mechanisms, but to what extent is currently unclear. Interestingly, islet beta-cell autoimmunity, which is associated with T1D, has also been observed in T2D subjects (Reed et al., 2021[262]), and systemic insulin resistance, islet amyloid and inappropriate glucagon activity, typically associated with T2D, can also manifest in T1D subjects (Bielka et al., 2024[38]; Bisgaard Bengtsen and Møller, 2021[39]; Denroche and Verchere, 2018[93]; Guo et al., 2021[133]; Westermark et al., 2017[329]). Latent autoimmune diabetes in adults (LADA) exhibits characteristics of both T1D and T2D, which clearly demonstrates that pathogenic mechanisms typically thought to be unique to each major form of diabetes can simultaneously manifest (Baker et al., 2013[26]; Buzzetti et al., 2020[55]; Ravikumar et al., 2023[259]). A similar situation occurs in youth, known as latent autoimmune diabetes in youth (LADY) (Cheng et al., 2022[65]; Pampapathi et al., 2024[235]). New, more effective therapeutic strategies could be developed by further understanding how the various pathologies can overlap mechanistically in T1D and T2D, and the associated implications this has on the disease severity/prognosis.

### Latent autoimmune diabetes in adults and youth

LADA is a form of diabetes that shares characteristics of both T1D and T2D (Ravikumar et al., 2023[259]). LADA diagnosis requires individuals to be over 30 years, exhibit milder T1D symptoms at diagnosis, not need insulin treatment for > 6 months after diagnosis and be positive for at least one of the T1D-associated autoantibodies (Buzzetti et al., 2020[55]; Ravikumar et al., 2023[259]). Given that these individuals typically present with symptoms more akin to T2D, they are often misdiagnosed as T2D (O'Neal et al., 2016[229]; Ravikumar et al., 2023[259]). A recent study estimated that 8.9 % of the global diabetic population had LADA (Ramu et al., 2023[258]). A previous study found a very similar observation, with 9.2 % of the T2D population and 0.6 % of the total population having LADA (Qi et al., 2011[254]). It could be argued that LADA is a milder form of T1D, given that autoantibodies are present and subjects typically progress sooner to requiring insulin treatment than T2D individuals (Sørgjerd et al., 2021[291]; Zhou et al., 2024[352] ). Although the progression to insulin dependence can vary greatly between LADA subjects, from months to many years (Sørgjerd et al., 2021[291]). However, an important distinguishing feature of LADA is that individuals exhibit insulin resistance (Chiu et al., 2007[67]; Ravikumar et al., 2023[259]), which implies similar pathology to T2D, despite the reduced frequency of obesity and metabolic syndrome in the LADA population (Buzzetti et al., 2020[55]; Ravikumar et al., 2023[259]; Zhou et al., 2024[352]). The risk of developing LADA has been associated with being overweight and adopting sedentary lifestyles, similar to T2D, but genetic predisposition similar to T1D has also been documented (Sørgjerd et al., 2021[291]). Hence, LADA has been referred to as type 1.5 diabetes, given the shared pathological features and risk factors between both of the major forms of diabetes (Ravikumar et al., 2023[259]). Interestingly, a similar situation to LADA occurs with individuals aged between 15 and 30, known as LADY; these subjects also do not require insulin treatment for > 6 months after diagnosis (Pampapathi et al., 2024[235]). Studies have found that 7.9-9 % of phenotypic T2D subjects in this age range test positive for islet autoantibodies associated with T1D, and this is associated with reduced islet beta-cell function and lower insulin resistance (Cheng et al., 2021[66], 2022[65]).

### Autoimmunity in T2D

Over recent years, it has become increasingly evident that people with T2D exhibit islet beta-cell autoimmunity usually associated with T1D and LADA/LADY (Ravikumar et al., 2023[259]; Reed et al., 2021[262]). Studies have demonstrated that islet-reactive cytotoxic T cells and islet beta-cell autoantibodies are present in a subset of T2D subjects (Itariu and Stulnig, 2014[157]). The prevalence of islet autoantibodies has been estimated to be between 5-30 % in the T2D population by studies (Brooks-Worrell et al., 2013[49]). A study conducted in 2000 found that 12 % of elderly T2D elderly individuals were positive for either GADA or IA-2A, and the presence of these autoantibodies was associated with impairment of acute-phase insulin secretion, revealed by an oral glucose tolerance test (Pietropaolo et al., 2000[246]). More recently, a study found that 11 % of the study population diagnosed with T2D had one or more of GADA, IAA, or ZnT8A, and these individuals presented with worse glycaemic control in comparison to the T2D autoantibody-negative population (Moosaie et al., 2021[215]). To further support the notion that the presence of islet autoantibodies exacerbates disease phenotype in T2D, another study found that a much higher percentage of autoantibody-positive participants were using insulin in comparison to autoantibody-negative individuals (Lomeli et al., 2025[192]). Although it is unknown if the participants in these studies were positive for any islet autoantibodies when they were diagnosed (Brooks-Worrell et al., 2013[49]; Lomeli et al., 2025[192]; Moosaie et al., 2021[215]; Pietropaolo et al., 2000[246]); if they were, then they could have met the criteria for a diagnosis of T1D, LADA or LADY. Any subsequent development of islet autoantibodies in established T2D subjects warrants consideration if they should then be additionally diagnosed with T1D, LADA or LADY, or be classified as having 'double diabetes', especially if their diabetic phenotype has worsened in tandem; this is a plausible scenario, as studies have demonstrated that detection of humoral islet autoimmunity is associated with worse glycaemic control and increased likelihood of dependency on insulin treatment (Brooks-Worrell et al., 2013[49]; Lomeli et al., 2025[192]; Reed et al., 2021[262]). Surprisingly, one study found that islet-reactive T cells were present in > 60 % of 36 phenotypic T2D subjects, who were all obese and had no history of ketoacidosis or insulin treatment, and > 30 % were also positive for at least one islet autoantibody (Brooks-Worrell et al., 2011[51]). The presence of islet-reactive cytotoxic T cells correlated with diminished islet beta-cell function in this study, but autoantibody presence alone did not. The cytotoxic T cell reactivity to ≥ 4 islet autoantigens was considered positive for T1D-associated cytotoxic T cell autoimmunity here. Interestingly, only 7/36 subjects in this study tested negative for any T cell reactivity to islet antigens, with 7/36 testing positive for 2 or 3, but not considered positive for T cell T1D-associated autoimmunity.

A subsequent study found that phenotypic T2D subjects who tested positive for islet-reactive T cells had improved islet beta-cell function with rosiglitazone treatment, which coincided with down-regulation of islet-specific T cell responses (Brooks-Worrell and Palmer, 2013[50]). This further demonstrated that islet-reactive T cells promote islet beta-cell pathology in T2D. Seemingly healthy controls can test positive for T cell reactivity to islet antigens, even multiple, and more than T1D subjects, but it is currently unclear why/how they do not have the T1D phenotype (Herold et al., 2009[147]). Confusingly, a subset of T1D subjects here did not test positive for any T cell reactivity to islet antigens, but it is possible they could be positive for antigens not tested in that study. A more recent, larger-scale study found that cellular islet autoimmunity was present, with reactivity to ≥ 4 islet autoantigens, in 41.3 % of participants with T2D currently on metformin treatment, and humoral autoimmunity, positivity for GADA, IA-2A and ZnT8A, was present in 13.5 % (Brooks-Worrell et al., 2022[48]). Both humoral and cell-mediated autoimmunity were present in 5.3 % of participants in that study. In the same study, many participants had reactivity to 1-3 autoantigens but did not meet ≥ 4 to be determined as T cell positive. Interestingly, T cell-mediated autoimmunity, but not humoral autoimmunity, was also associated with worse islet beta-cell function and glycaemic control. The observation here that humoral autoimmunity did not appear to worsen disease phenotype contradicts findings from other aforementioned studies (Lomeli et al., 2025[192]; Moosaie et al., 2021[215]; Pietropaolo et al., 2000[246]). Rodent studies have also generated evidence for autoimmunity being involved in T2D pathogenesis, as B lymphocyte-deficient New Zealand obese mice, an obese polygenic mouse model of obesity and T2D, did not develop T2D (Haskell et al., 2002[141]), which implies that B lymphocytes are essential for T2D manifestation in these mice. Another study found that B lymphocytes from T2D subjects were unable to secrete the anti-inflammatory cytokine IL-10 and hypersecreted IL-8, suggesting that these cells encourage inflammation and worsen the disease phenotype (Jagannathan et al., 2010[160]).

These observations clearly demonstrate that T1D-associated autoimmunity is present in T2D and has some impact on disease severity. Although they raise some important questions, such as why T2D individuals with T1D-associated cytotoxic T cells do not have the more severe disease phenotype of T1D and whether LADA/LADY diagnosis also needs to take into account if individuals are positive for these T cells as well as autoantibodies. By currently unclear mechanisms, T2D individuals' islet beta-cells are seemingly able to avoid the severe autoimmune destruction usually brought on by T1D-associated cytotoxic T cells. Perhaps the increased expression of HLA seen in T1D does not occur in this scenario (Richardson et al., 2011[267]). Discovery of new autoantigens associated with diabetic autoimmunity could raise the detection rates of islet beta-cell autoimmunity in all forms of diabetes, in both the pre-clinical and clinical phases. The transient nature of islet autoantibodies (Achenbach et al., 2005[2]; Phillip et al., 2024[245]; Sørgjerd, 2019[290]; Vehik et al., 2016[321]; Williams et al., 2022[333]) needs to be considered when screening T2D subjects, since if an individual tests negative, this does not mean they have not been positive for one of these autoantibodies before and/or they may develop positivity in the future. Detection of islet autoreactive T cells can also be difficult to monitor in individuals, as 3/36 subjects who initially tested negative were found to be positive in subsequent checks over six months (Brooks-Worrell et al., 2011[51]). The current notion that subjects need T cell reactivity to ≥ 4 islet autoantigens in order to be determined as having T cell T1D autoimmunity needs revision, as this may underestimate the presence of islet autoimmunity, as ideally, no positivity should be present in individuals with no T1D-associated pathology. The observations that seemingly healthy controls can have positivity for islet antigens, even multiple, > 4, does not mean that pathology is not present, as these individuals might be in the preclinical diabetic phase, and may not ever progress to clinical disease for currently unclear reasons. Further understanding the regulation and contribution of islet beta-cell autoimmunity to disease pathogenesis and severity, as well as improving its detection, will likely enable the development of more effective therapeutic strategies for T1D, T2D and LADA/LADY.

### Altered glucagon secretion in T1D

Inappropriate glucagon secretion is well known to contribute to the disease phenotype in T2D (Reed et al., 2021[262] ). However, in recent years, inappropriate glucagon activity has also been found in T1D and has attracted scientific attention (Bisgaard Bengtsen and Møller, 2021[39]; Guo et al., 2021[133]). Similar to what is observed in T2D, T1D subjects exhibit inappropriate/excessive postprandial hyperglucagonemia, which further exacerbates hyperglycaemia (Guo et al., 2021[133]; Zhang et al., 2024[345]). One study found that postprandial plasma glucagon levels increased by 160 % five years after T1D diagnosis (Fredheim et al., 2015[119]). Another study found that T1D individuals with high postprandial glucose tended to have high postprandial glucagon levels (Urakami et al., 2011[317]). In contrast, during hypoglycaemia in some, typically long-duration T1D subjects, glucagon activity is absent and unable to raise blood glucose (Bisgaard Bengtsen and Møller, 2021[39]). Therefore, in T1D, excessive glucagon activity worsens the disease phenotype during the postprandial period and diminished glucagon activity is unable to raise blood glucose during hypoglycaemia (Bisgaard Bengtsen and Møller, 2021[39]; Guo et al., 2021[133]). Given the findings from various studies (Bisgaard Bengtsen and Møller, 2021[39]; Guo et al., 2021[133]; Reed et al., 2021[262]), T1D and T2D should be viewed as bihormonal disorders; further understanding how altered islet alpha-cell activity and glucagon secretion manifests mechanistically in these disorders will likely enable new therapeutic options.

### Insulin resistance in T1D

Systemic insulin resistance is a well-established pathological feature of T2D (Reed et al., 2021[262]). However, insulin resistance has also been observed in people with T1D, and these individuals are classified as having 'double diabetes' (Bielka et al., 2024[38]). Insulin resistance manifests in an estimated 25-30 % of T1D individuals (Chaudhary et al., 2025[63]). It is not surprising that T1D subjects can develop systemic insulin resistance post-diagnosis due to complications associated with the intense insulin treatment and associated weight gain and/or if they adopt excessive diets/unhealthy lifestyles (Apostolopoulou et al., 2025[16]; Bielka et al., 2024[38]). The pathology that promotes insulin resistance in overweight T1D and T2D subjects is likely similar. Curiously, though, insulin resistance has also been observed in lean T1D subjects (Donga et al., 2013[99]), which suggests that the pathogenesis of insulin resistance cannot be attributed only to excessive weight gain. Understanding mechanistically how insulin resistance manifests in non-overweight T1D and T2D subjects may lead to the development of new therapeutic strategies. Insulin resistance manifestation during the pre-clinical phase of T1D has also been identified to encourage progression to the clinical phase, independently of other risk markers such as weight and age (Apostolopoulou et al., 2025[16]; Fourlanos et al., 2004[118]). These observations suggest that insulin resistance is also important in T1D pathogenesis as well as T2D. If induction of insulin resistance occurs in overweight and non-overweight individuals via similar mechanisms and has similar clinical implications in both major forms of diabetes before and after diagnosis, then this would further challenge the current distinction between them.

### Amyloid in T1D

IAPP plaques are present in the islets of the majority of individuals with T2D and intracellular oligomers of IAPP are thought to promote islet beta-cell dysfunction in this disease (Reed et al., 2021[262]). Interestingly, one study found that 11 % of recently diagnosed T1D subjects had abnormally high plasma concentrations of IAPP (Paulsson et al., 2014[239]). A subsequent study then found that 2 out of 6 recent-onset T1D pancreas biopsies had both intra- and extracellular amyloid present, associated with dying islet beta-cells (Westermark et al., 2017[329]). Another more recent study identified that islet amyloidosis was present in all three donors with T1D (Beery et al., 2019[33]). Therefore, given these observations, it seems that islet amyloid is also present in at least a subset of T1D subjects and could contribute to disease pathology in a similar manner to T2D. Evidence has emerged that IAPP and its precursor forms contain several epitopes that act as autoantigens and can activate T cells in NOD mice and human T1D (Denroche and Verchere, 2018[93]), implying that islet IAPP encourages autoimmunity in the islets of T1D subjects. Genetic deletion of IAPP did not alter diabetes progression in NOD mice, indicating that this is not an essential autoantigen in T1D (Baker et al., 2013[26]). Developing treatments that prevent/degrade islet amyloid accumulation may assist in alleviating the T1- and T2D phenotypes by improving islet beta-cell function.

## Alzheimer’s Disease

AD is a progressive, irreversible neurodegenerative disorder characterised by a gradual decline of cognitive function, including memory loss, impaired ability to function, and behavioural changes (Breijyeh and Karaman, 2020[47]; Kamatham et al., 2024[171]; Safiri et al., 2024[271]). An estimated 50 million people have dementia globally and this number is expected to triple by 2050 (Aranda et al., 2021[17]). AD pathogenesis is complex, involving currently mechanistically unclear interactions between genetics and environment that initiate the disease (Breijyeh and Karaman, 2020[47]; Kamatham et al., 2024[171]; Safiri et al., 2024[271]). Pharmacological treatments are available for AD, which alleviate the symptoms but do not slow disease progression (Safiri et al., 2024[271]). Cholinesterase inhibitors, which act to decrease acetylcholine breakdown and preserve communication between neurons during AD pathogenesis, and memantine, which acts as an NMDA receptor antagonist, reducing the receptors' chronic overstimulation by excess glutamate, which manifests during AD pathology, are the standard medications to alleviate symptoms of this disease (Kamatham et al., 2024[171]; Safiri et al., 2024[271]; Thangwaritorn et al., 2024[306]; Wu and Fuh, 2025[337]). In recent years, anti-amyloid immunotherapy has demonstrated efficacy in slowing cognitive decline in early-stage AD, and the FDA has approved the use of monoclonal antibodies such as lecanemab for treatment (Thangwaritorn et al., 2024[306]; Wu and Fuh, 2025[337]). This anti-amyloid immunotherapy is the first therapeutic strategy for AD that has been documented to delay disease progression (Elamin et al., 2025[110]; Wu and Fuh, 2025[337]). Research has revealed that AD is a multifactorial disease, and although much progress has been made in understanding certain aspects of this disease, such as its aetiology and pathogenesis, much remains to be elucidated, and more effective therapeutic strategies are needed (Breijyeh and Karaman, 2020[47]; Kamatham et al., 2024[171]; Safiri et al., 2024[271]; Wang et al., 2018[324]).

### Epidemiology

**Figure 4:**
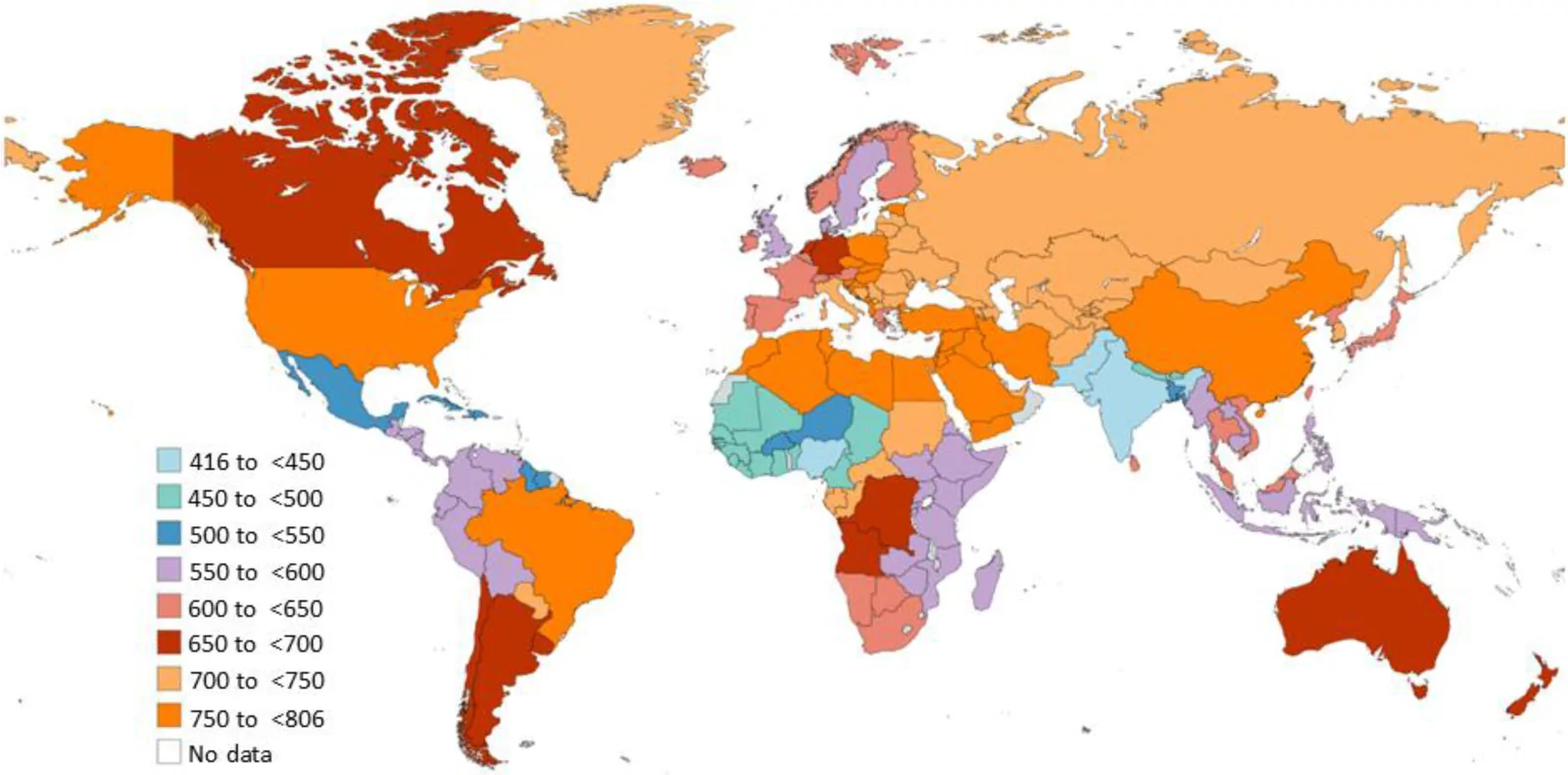
Comparing the global distribution of age-standardised prevalence of AD per 100,000 between countries in 2019. This figure was generated with data taken from (Safiri et al., 2024).

Overall, the global incidence and prevalence of AD have increased over recent decades (Li et al., 2022[186]; Safiri et al., 2024[271]). Globally, the number of dementia patients has increased by an estimated 117 %; in 2016, 43.8 million versus 20.3 million in 1990 (GBD 2016 Dementia Collaborators, 2019[123]). Incidence and prevalence rates increase with increasing age in dementia, and therefore, given the global rise in the ageing population and increased life expectancy, the disease burden is expected to massively increase in the future (Li et al., 2022[186]). By 2050, it is estimated that there will be 152 million people living with dementia globally (GBD 2019 Dementia Forecasting Collaborators, 2022[124]). Given that AD is the most common form of dementia and accounts for an estimated 60-80 % of all dementia cases (Kamatham et al., 2024[171]), it is a major global healthcare burden and a concern for the future. The presence of dementia in 2019 varied between regions/nations, with East Asia, North Africa and the Middle East all reporting an estimated > 700 cases and South Asia and Western Sub-Saharan Africa reporting an estimated < 500 cases per 100,000 (Safiri et al., 2024[271]). Interestingly, marked increases in dementia prevalence have been found in 2021 compared to 1990 in some countries (Safiri et al., 2024[271]; Zhi et al., 2025[349]), with the total prevalence rate increasing in China from 342.1/100,000 in 1990 to 1194.2/100,000 in 2021 (Zhi et al., 2025[349]). After age standardisation, the dementia prevalence rate in China increased by 30.6 % for females and 27.3 % for males in 2021. Figure 4(Fig. 4) (References in Figure 4: Safiri et al., 2024[271]) shows how the global prevalence of AD varied in 2019.

### Aetiology

AD has a multifactorial and incompletely understood aetiology, involving genetics, ageing, the presence of other diseases and environmental factors (Breijyeh and Karaman, 2020[47]; Armstrong, 2019[18]; Safiri et al., 2024[271]). Ageing is considered the primary risk factor for AD, with an estimated 1 in 10 individuals aged ≥ 65 having the disease (Liu et al., 2024[190]). The incidence of AD doubles every 5 years after age 65, with an estimated one-in-three individuals aged over 85 developing the disease (Isik, 2010[156]). Approximately 95 % of AD patients are diagnosed after age 65 (Loi et al., 2023[191]), known as late-onset AD (LOAD), which clearly demonstrates an old age association with AD risk. However, AD can manifest in individuals as young as 40 and the prevalence and incidence of dementia manifesting in individuals under 65, known as early-onset AD (EOAD), has increased in recent decades (He et al., 2025[144]). Family history is the second greatest risk factor for AD, with an estimated 35-60 % of EOAD individuals having a first-degree relative with dementia (Safiri et al., 2024[271]). Twin and family studies indicate that genetic factors play a role in an estimated 80 % AD cases (Gatz et al., 2006[122]). EOAD is associated with variants of the amyloid precursor protein (APP), presenilin-1 (PSEN1) and presenilin-2 (PSEN2) genes, which account for 18-50 % of familial EOAD cases (Cai et al., 2015[56]; Tanzi, 2012[302]). LOAD risk is associated with the e4 allele of the apolipoprotein E APOE gene (Tanzi, 2012[302]), and homozygous subjects for e4 have an 8-12 fold increased risk of developing AD (Altmann et al., 2024[11]). These four genes account for 30-50 % of AD inheritability (Tanzi, 2012[302]). Although GWAS have identified more than 80 susceptibility loci (Andrews et al., 2023[15]), reported odds ratios for variants are modest, typically ranging from 1.1 to 2.4 (Andrews et al., 2023[15]; Shen and Jia, 2016[283]). Environmental and behavioural factors have also been identified to increase AD risk, including smoking, excessive alcohol consumption, sedentary lifestyle, and excessive diet/obesity (Safiri et al., 2024[271]). Lifetime smoking was associated with a 70 % greater risk of AD development (Barnes and Yaffe, 2011[29]; Durazzo et al., 2014[104]) and more physically active subjects had a 45 % reduced risk (Hamer and Chida, 2009[137]). Several studies have found that midlife obesity increases the risk of dementia and AD by ≥ 60 % (Albanese et al., 2017[8]; Kivipelto et al., 2005[175]). Health conditions such as cardiovascular disease (CVD) and diabetes also increase the risk of developing AD. Both T1D and T2D raise the risk of developing AD by around 50 % (Cholerton et al., 2016[68]; Li et al., 2025[184]). Hypertension and associated risk factors account for 2-9.6 % of dementia cases globally (Brain et al., 2023[46]). One study found that after 5.4 years, participants with CVD had a higher risk of dementia and AD than those without CVD (Newman et al., 2005[217]).

### Pathogenesis

AD pathogenesis involves multiple intertwined mechanisms, including accumulation of amyloid-beta (Aß) plaques, neurofibrillary tangles (NFTs), neuroinflammation, synaptic dysfunction, and neuronal loss/atrophy (Kamatham et al., 2024[171]). AD is a prominent protein-conformational disease, largely induced by incorrect processing, altered conformations and polymerisation of normally physiologically beneficial proteins (Kamatham et al., 2024[171]; Tiwari et al., 2019[308]). AD is characterised histopathologically by extracellular aggregates of Aβ plaques and intracellular aggregations of NFTs, composed of hyperphosphorylated microtubule-associated tau (DeTure and Dickson, 2019[94]; Tiwari et al., 2019[308]).

Amyloid formation is thought to initiate AD pathology (Fan et al., 2020[112]; Kamatham et al., 2024[171]; Tiwari et al., 2019[308]). Aß plaques are thought to first manifest in the basal, temporal, and orbitofrontal neocortex regions of the brain, and as the disease progresses, these plaques also form throughout the neocortex, hippocampus, amygdala, diencephalon and basal ganglia, cerebellar cortex, striatum and thalamus (Planche et al., 2022[249]; Tiwari et al., 2019[308]). Aß aggregation is thought to cause neuronal damage/death by several mechanisms, such as excitotoxicity, synaptic disruption, oxidative stress, impaired intracellular transport and mitochondrial dysfunction (Kamatham et al., 2024[171]; Tiwari et al., 2019[308]; Zhang et al., 2023[347]). Interestingly, several studies have found that cognitively unimpaired elderly subjects can have significant Aß deposition, while others have found that a positive correlation exists between deposition and cognitive decline and dementia severity (Aizenstein et al., 2008[6]; Cummings and Cotman, 1995[81]; Dickson et al., 1992[95]; Nielson et al., 1996[220]; Wang et al., 2024[325]). It is currently unclear why Aß deposition promotes pathology in some but not others, and how the aberrant processing of amyloid precursor protein by Β-site APP cleavage enzyme 1 (BACE1) and γ-secretases to form Aß is initiated in the disease state remains mechanistically unclear (Aizenstein et al., 2008[6]; Jansen et al., 2015[162]; Kamatham et al., 2024[171]; Tiwari et al., 2019[308]). NFTs containing hyperphosphorylated tau are thought to form after Aß deposition, as the pathological Aß deposits cause kinases to be released, which then hyperphosphorylate tau and thereby cause it to be oligomerised (Kamatham et al., 2024[171]; Tiwari et al., 2019[308]). This then leads to the aggregation of insoluble tau filaments forming NFTs in neurons, leading to loss of signalling between neurons and impaired cytoskeletal function (Jiang et al., 2025[169]; Tiwari et al., 2019[308]). The tau aggregations are abundant components of NFTs found in the brains of people with AD (Kamatham et al., 2024[171]). Additionally, AD-associated tau has been suggested to act as a prion, whereby it induces misfolding of normal tau in neighbouring neurons, leading to progressive tau pathology across the brain (Clavaguera et al., 2015[69]; Kamatham et al., 2024[171]).

The Aß plaques and NFTs are also speculated to drive chronic neuroinflammation, which further induces neuronal damage and in turn, encourages further Aß deposition (Kamatham et al., 2024[171]; Tiwari et al., 2019[308]; Wong-Guerra et al., 2023[335]). The ongoing neuronal toxicity due to these plaques and NFTs induces migration of microglia and astrocytes into the affected areas of the brain, with the intention of these cells to remove the plaques (Deng et al., 2024[92]; Kamatham et al., 2024[171]). Aß plaques are known to activate toll-like receptors directly on microglia, causing the release of proinflammatory cytokines, and so these plaques directly promote neuroinflammation (Heneka et al., 2015[145]; Tiwari et al., 2019[308]). In AD, the immune response is thought to be pathological overall and fails to adequately clear the environment of plaques and NFTs, resulting in a chronic inflammatory state, which further promotes neurotoxicity (Kamatham et al., 2024[171]; Tiwari et al., 2019[308]; Wong-Guerra et al., 2023[335]). Mitochondrial dysfunction is also induced directly by the accumulation of Aß peptides (Kamatham et al., 2024[171]). Aß intracellular oligomers can accumulate within the inter-mitochondrial membrane, leading to reduced ATP synthesis and increased ROS production (Anandatheerthavarada et al., 2003[14]; Averchuk et al., 2023[23]; Kamatham et al., 2024[171]). Reduced ATP metabolically strains the neurons, and increased ROS levels promote cytotoxicity via oxidative stress (Kamatham et al., 2024[171]; Tiwari et al., 2019[308]). Aß-induced mitochondrial damage initiates a vicious cycle, as impaired mitochondrial function further promotes the Aß pathology (Anandatheerthavarada et al., 2003[14]). AD-associated tau impairs mitochondrial transport along neuronal axons, resulting in further oxidative stress and reduced energy production throughout the cell (Shahpasand et al., 2012[282]). In turn, mitochondrial dysfunction and oxidative stress then further encourage tau pathology (Du et al., 2022[102]). Additionally, oxidative stress also contributes to the formation of Aß plaques and NFTs, further exacerbating the neurodegeneration (Roy et al., 2023[269]).

**Figure 5:**
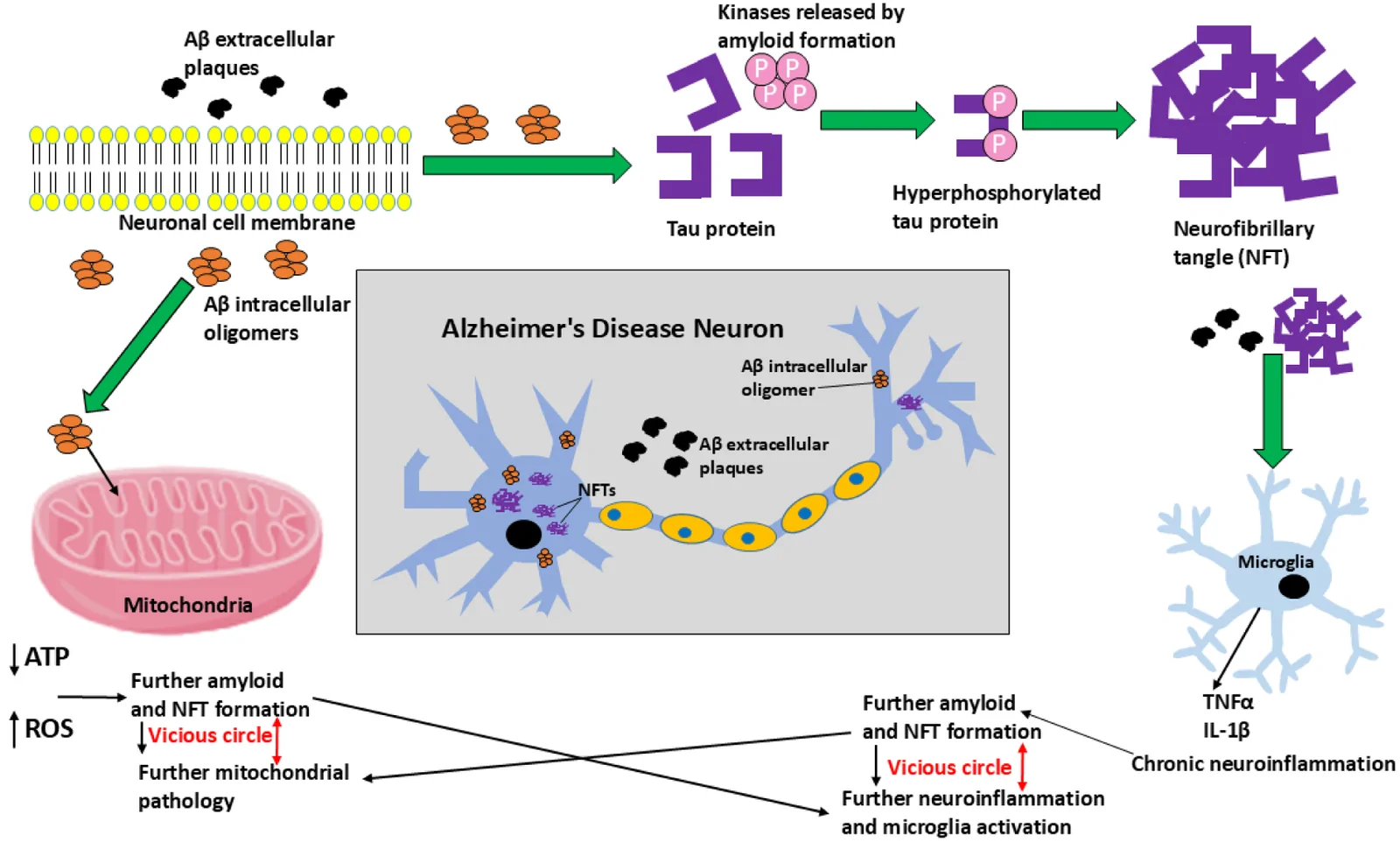
Overview of the pathogenesis of AD. Neuronal amyloid deposition is thought to initiate AD pathogenesis in the form of Aβ intracellular oligomers and extracellular plaques. This amyloid deposition then initiates tau hyperphosphorylation and formation of NFTs, as well as mitochondrial dysfunction resulting in increased ROS formation and reduced ATP. The mitochondrial dysfunction then further encourages amyloid and NFT formation, which in turn, further drives mitochondrial pathology, setting up a vicious circle. Chronic neuroinflammation is driven by the presence of extracellular Aβ plaques and NFTs, inducing activation of microglia. The chronic neuroinflammation then induces further amyloid and NFT formation, which further enhances neuroinflammation and microglial activation, setting up another vicious circle. All of these pathological mechanisms result in progressive loss of neuronal function, resulting in the clinical manifestation of AD, and the disease phenotype worsens with time post diagnosis due to ongoing neuronal pathology. This figure and the information in its legend are adapted from (Averchuk et al., 2023; Deng et al., 2024; DeTure and Dickson, 2019; Du et al., 2022; Jansen et al., 2015; Jiang et al., 2025; Kamatham et al., 2024; Planche et al., 2022; Roy et al., 2023; Shahpasand et al., 2012; Tiwari et al., 2019; Wong-Guerra et al., 2023; Zhang et al., 2023).

Thus, the formation of Aß plaques initiates a cascade of pathological events that gradually impair neuronal functioning and insidiously promote neurotoxicity (Kamatham et al., 2024[171]; Tiwari et al., 2019[308]). The Aß plaques, NFTs, neuroinflammation and mitochondrial dysfunction interact synergistically to induce progressive AD pathology during the pre-clinical and clinical phases (Kamatham et al., 2024[171]; Parnetti et al., 2019[237]; Tiwari et al., 2019[308]; Zhang et al., 2024[344]). After initiation of AD pathology, the rate of progression can vary greatly between subjects, with some individuals remaining in the pre-clinical asymptomatic phase for > 20 years (Caselli et al., 2020[61]; Jia et al., 2024[167]; Zhang et al., 2024[344]). Additionally, the duration of mild cognitive impairment, which manifests prior to AD diagnosis, and clinical AD also varies substantially between subjects (Zhang et al., 2024[344]). Not all individuals with mild cognitive impairment progress to AD, which is not always progressive and is sometimes reversible, and can be caused by a variety of factors, such as diabetes and depression (Dunne et al., 2020[103]; He et al., 2024[143]; Salemme et al., 2025[273]). Diagnosis of AD occurs through a combination of cognitive tests, blood tests, brain scans and physical examination, reviewed elsewhere (Bomasang-Layno and Bronsther, 2021[41]; Safiri et al., 2024[271]). AD progresses from the mild stage associated with memory loss, speech impairment and mood changes to the severe stage where patients' motor functions and day-to-day activities are impaired, and eventually patients become bedridden and need assistance with basic tasks such as eating (Clifford et al., 2024[71]; Kamatham et al., 2024[171]). Typically, patients survive 3-10 years after diagnosis, and life expectancy is influenced by age at onset, in addition to other factors such as existing comorbidities (Brück et al., 2025[53]; Zanetti et al., 2009[342]). Death in AD subjects usually arises from the manifestation of other age-related diseases and/or from post-diagnosis complications such as pneumonia (Manabe et al., 2019[201]; Todd et al., 2013[309]; Tolppanen et al., 2020[310]). Figure 5(Fig. 5) (References in Figure 5: Averchuk et al., 2023[23]; Deng et al., 2024[92]; DeTure and Dickson, 2019[94]; Du et al., 2022[102]; Jansen et al., 2015[162]; Jiang et al., 2025[169]; Kamatham et al., 2024[171]; Planche et al., 2022[249]; Roy et al., 2023[269]; Shahpasand et al., 2012[282]; Tiwari et al., 2019[308]; Wong-Guerra et al., 2023[335]; Zhang et al., 2023[347]) summarises AD pathogenesis.

## Alzheimer’s Disease Is Type 3 Diabetes

The notion that AD is T3D was generated due to the detection of insulin resistance and/or impaired insulin signalling in patients' brains, which could account for the disease pathogenesis (Jash et al., 2020[165]; Peng et al., 2024[240]). Historically, the brain was thought to be insensitive to insulin, but evidence continues to emerge that this is not the case, as glucose transporter 4 (GLUT4), insulin-like growth factor-1 (IGF-1) and other insulin-sensitive receptors are present on the surface of neurons and glial cells (Arrieta-Cruz and Gutiérrez-Juárez, 2016[19]; Ebrahimpour et al., 2020[106]; Peng et al., 2024[240]). Insulin action is also important for neuronal circuitry formation, synaptic plasticity, neuronal survival, neurotransmitter expression, signal transduction and memory (Ebrahimpour et al., 2020[106]). Markers of insulin resistance have been found in AD patient brains and neuronally derived exosomes, although some studies have not found this (Craft et al., 2020[78]; Thambisetty et al., 2013[305]). AD experimental models have also observed impaired insulin signalling (Ferreira et al., 2018[114]). The most convincing evidence to date that AD brains exhibit insulin resistance, even in the absence of diabetes, comes from post-mortem indexes of brain insulin resistance (Arvanitakis et al., 2020[20]; Leclerc et al., 2022[182]; Talbot et al., 2012[301]). Thus, the manifestation of AD insulin resistance is not dependent on T2D. To further demonstrate AD's independence from T2D, brains of people with diabetes do not exhibit an increased frequency of amyloid plaques associated with AD (Janson et al., 2004[163]). Interestingly, insulin and IGF-1 were also shown to be downregulated in the brains of AD patients (Steen et al., 2005[295]). In that study, insulin gene expression was detected in the hippocampus and hypothalamus of human post-mortem brain tissue, with insulin mRNA expression being 4-fold lower in the hippocampus and 2-fold lower in the hypothalamus of AD patients compared to corresponding samples from brains exhibiting normal ageing. However, the physiological relevance of any locally produced insulin and IGF-1 in the brain is still unclear, given that insulin and IGF-1 can cross the blood-brain barrier from the circulation, so associated signalling in the brain is not typically thought to be dependent on any local production (Cao et al., 2023[58]; Nguyen et al., 2020[219]). It is reasonable to assume that if insulin is produced locally in the human brain, which is currently an area of debate (Dakic et al., 2023[85]), then it likely plays an important physiological role; therefore, insulin deficiency in AD brains could be another reason to view this disease as T3D. Progressive AD Braak stage-dependent reductions in insulin, IGF-1 and IGF-2 receptor expression have been reported, suggesting that the loss of these receptors worsens in tandem with progressive AD pathology (de la Monte and Wands, 2008[90]). Further evidence that impaired insulin signalling is central to AD was demonstrated by improved cognitive performance in experimental models and humans with AD or mild cognitive impairment, after treatment with insulin sensitiser agents or intranasal insulin (de la Monte et al., 2006[89]; de la Monte and Wands, 2008[90]).

**Figure 6:**
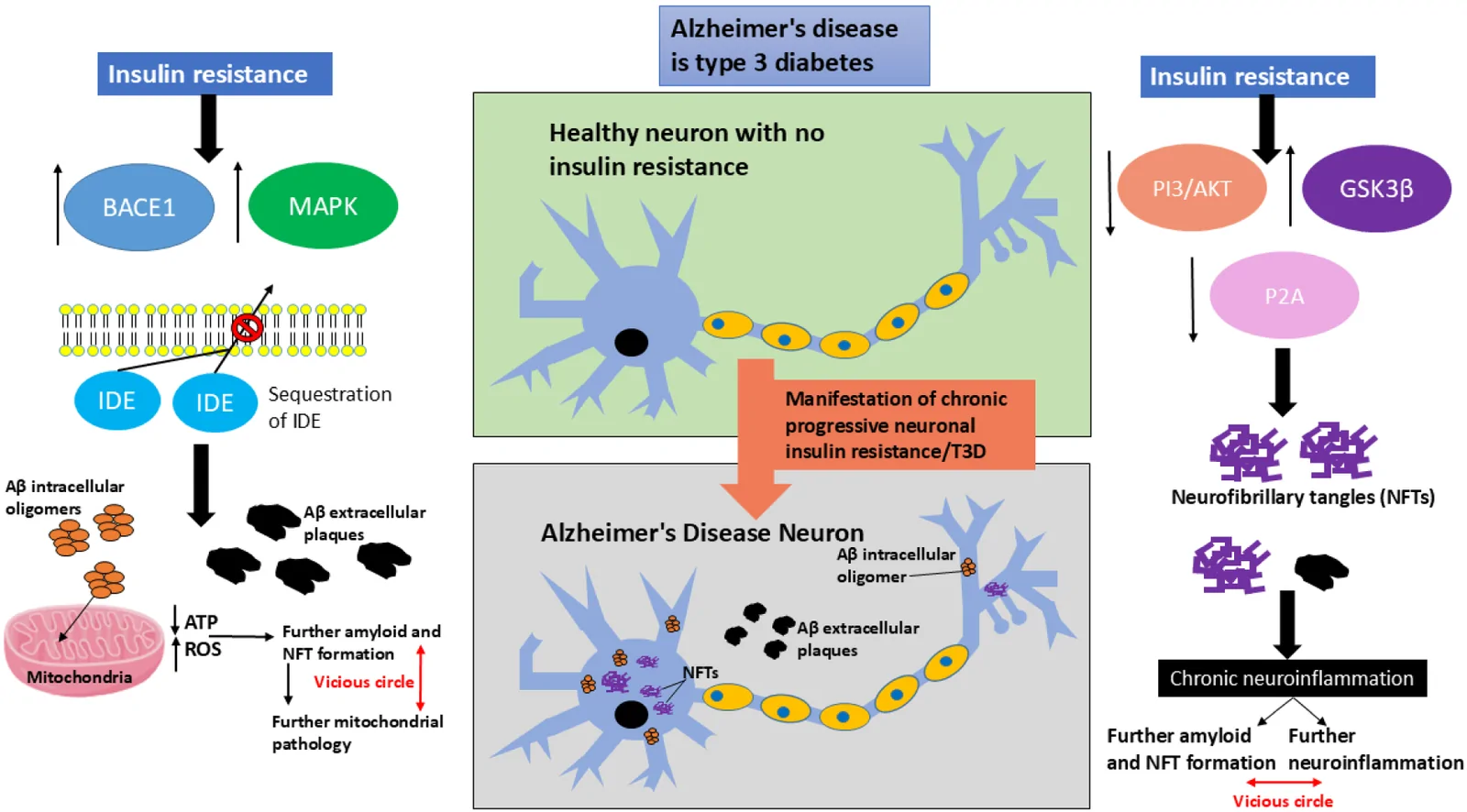
Overview of how the manifestation of insulin resistance in neuronal cells in the brain could mechanistically initiate AD/T3D pathogenesis. Upon manifestation of chronic progressive neuronal insulin resistance, which occurs via currently unclear mechanisms, increased BACE1 and MAPK pathway activity, as well as sequestration of IDEs, result in the formation of Aβ amyloid, which results in the formation of NFTs, mitochondrial pathology and neuroinflammation. Insulin resistance also directly promotes NFT formation via reducing activation of the PI3K/AKT pathway and promoting activation of GSK3β. Insulin resistance additionally results in P2A inhibition. The NFT formations then further encourage neuroinflammation and neurotoxicity. All of these pathological mechanisms result in progressive loss of neuronal function, which correlates with progressive worsening insulin resistance over time in the T3D pathogenesis model. This figure and the information in its legend are adapted from (Atabi et al., 2025; Gratuze et al., 2017; Haque and Nazir, 2014; Jash et al., 2020; Jia et al., 2024; Kamatham et al., 2024; Milionis et al., 2014; Peng et al., 2024; Qiu and Folstein, 2006).

Brain insulin resistance and hyperinsulinemia are speculated to encourage/initiate the formation of Aß peptides and plaques by augmenting BACE1 expression and stimulating the mitogen-activated protein kinase signalling pathway (Gasparini et al., 2001[121]; Peng et al., 2024[240]). Insulin resistance additionally reduces the activation of the phosphatidylinositol 3-kinase/protein kinase B (PI3K/AKT) pathway, which results in activation of one of the kinases, glycogen synthase kinase-3 beta (GSK3β), involved in tau phosphorylation (Peng et al., 2024[240]). In T3D, it is suggested that the increased GSK3β activity, due to insulin resistance, mediates tau hyperphosphorylation, which then results in the formation of NFTs (Milionis et al., 2014[213]). Insulin resistance also results in protein phosphatase 2A (P2A) inhibition, which further encourages tau hyperphosphorylation and subsequent formation of NFTs (Gratuze et al., 2017[131]). A further mechanism by which insulin resistance promotes AD pathology during hyperinsulinemia is by causing sequestration of insulin-degrading enzyme (IDE), which is involved in Aß degradation, resulting in impaired Aß clearance and further pathological amyloid formation (Haque and Nazir, 2014[138]; Peng et al., 2024[240]; Qiu and Folstein, 2006[256]). One study found that rats treated with excess insulin to induce insulin resistance had significantly reduced Aß clearance (Benedict and Grillo, 2018[36]). The NFTs and amyloid formations then further encourage neurotoxicity by aforementioned mechanisms, such as mitochondrial dysfunction, increased ROS generation and encouraging chronic neuroinflammation (Atabi et al., 2025[21]; Kamatham et al., 2024[171]; Peng et al., 2024[240]). GLUT4 translocation is also impaired during insulin resistance, which results in reduced glucose uptake by neurons, and the subsequent metabolic strain is likely another mechanism that promotes neurotoxicity (Atabi et al., 2025[21]). Over time, this pathology insidiously progresses until symptoms manifest, resulting in a diagnosis of AD, which is suggested to be T3D if the pathology is driven by brain insulin resistance (Atabi et al., 2025[21]; Caselli et al., 2020[61]; Jia et al., 2024[167]). Figure 6(Fig. 6) (References in Figure 6: Atabi et al., 2025[21]; Gratuze et al., 2017[131]; Haque and Nazir, 2014[138]; Jash et al., 2020[165]; Jia et al., 2024[167]; Kamatham et al., 2024[171]; Milionis et al., 2014[213]; Peng et al., 2024[240]; Qiu and Folstein, 2006[256]) summarises how insulin resistance may induce AD pathology.

It is firmly established that pancreatic islet dysfunction is central to T1D and T2D manifestation (Addissouky et al., 2024[4]; Reed et al., 2021[262]). However, pancreatic islet dysfunction may also be involved in AD pathology. Interestingly, hyperinsulinemia, which typically manifests to compensate for systemic insulin resistance prior to T2D, has been shown to increase the risk of AD and mild cognitive decline (Amin et al., 2023[13]; Luchsinger et al., 2004[198]). AD risk was doubled in individuals with hyperinsulinemia in one study, with the risk being higher in subjects without diabetes (Luchsinger et al., 2004[198]). These observations suggest that AD is encouraged by hyperinsulinemia independently of T2D. Additionally, glucagon levels in women with AD have been reported to be increased, although these patients did not have diabetes or issues with glucose tolerance (Vankova et al., 2021[320]). Another study found that individuals with AD or mild cognitive impairment had elevated serum levels of glucagon and ghrelin (de la Monte et al., 2019[88]). It is currently unclear what role increased glucagon/ghrelin via direct/indirect mechanisms may have in AD pathology and this has not been well studied. IAPP monomers and small oligomers released from the pancreatic islets can cross the blood-brain barrier and co-deposit with Aß in AD brains, which likely worsens the pathology (Bortoletto and Parchem, 2023[43]; Raimundo et al., 2020[257]). Interestingly, IAPP deposition in the brain has been shown to impair function independently of Aß (Srodulski et al., 2014[293]), and these deposits have been observed in the brains of people with AD, even in the absence of diabetes (Oskarsson et al., 2015[233]; Raimundo et al., 2020[257]). IAPP has also been shown to promote Aß aggregation in several studies, which would promote AD pathology (Ge et al., 2018[125]). More recent studies have also found that IAPP co-localises with tau in AD patients and encourages the formation of highly neurotoxic tau oligomer species (Bortoletto and Parchem, 2023[43]; Zhang et al., 2022[343]). Additionally, IAPP deposits have been shown to induce neuroinflammation and microglia clustering around these deposits in rodents (Bortoletto and Parchem, 2023[43]; Foguel and Azevedo, 2018[115]), indicating that these deposits may promote the neuroinflammation observed in AD. Several studies have also shown that human IAPP induces cell death in various neuronal populations via several mechanisms, such as increasing membrane permeabilisation and subsequent increased ROS generation (Bortoletto and Parchem, 2023[43]; Zhang et al., 2020[346]). Blocking of IAPP aggregation in *in vitro* studies has also been shown to prevent neuronal cell death (Yu et al., 2015[340]). Given the association of T2D and T1D with the presence of islet amyloid (Gupta and Leahy, 2014[134]; Smith et al., 2022[289]; Westermark et al., 2017[329]), it is reasonable to assume that during progressive diabetic pancreatic pathology, the ongoing islet beta-cell damage/death likely results in pathological IAPP oligomers and misfolded forms being released into the circulation, which could then promote AD pathology. Hyperamylinemia, associated with hyperinsulinemia during insulin resistance, as IAPP is co-secreted with insulin, has also been suggested to promote IAPP aggregation and misfolding, which then contributes to AD pathology (Raimundo et al., 2020[257]; Rehn et al., 2024[264]). Thus, given the emerging evidence, crosstalk between the pancreas and the brain needs to be taken into consideration when attempting to further understand the pathogenesis of AD (Bortoletto and Parchem, 2023[43]; Raimundo et al., 2020[257]). Interestingly, a recent study found that fibroblast growth factor 23 (FGF23), secreted by the Min6 pancreatic beta-cell line, attenuated PC12 cell death in response to Aß treatment (Yazawa et al., 2025[338]). The observations from this study imply that pancreatic islet secretion may also play an important role in promoting neuronal cell survival in the brain during healthy circumstances and AD pathogenesis in humans. It is reasonable to assume that during diabetic pathology, the reduced physiologically desirable activity of islet beta-cells may also promote AD pathogenesis via reduced secretion of neuroprotective factors and insulin, in addition to promotion of brain amyloid formation via pathological IAPP brain deposition.

T1D and T2D are well-known risk factors for AD, with each raising the risk by around 50 % (Cholerton et al., 2016[68]; Jeong et al., 2025[166]; Li et al., 2025[184]). Studies have found that the majority, around 80 %, of AD patients have T2D or impaired glucose tolerance (Barbagallo and Dominguez, 2014[28]). One study found that 35 % of AD patients had diabetes and 46 % had glucose intolerance (Convit, 2005[75]). Interestingly, one study found that AD patients had nearly double the risk of developing T2D and impaired glucose tolerance (Janson et al., 2004[163]). Recently, another study found that diabetes accelerates progression from mild cognitive impairment within one year of its diagnosis to AD (Ding et al., 2024[96]). These observations demonstrate that the pathology associated with diabetes can contribute to AD development and vice versa. To consider AD as T3D could be argued to be justifiable, given the evidence indicating that brain insulin resistance is an important component of its pathogenesis (de la Monte and Wands, 2008[90]; Nguyen et al., 2020[219]; Peng et al., 2024[240]). However, it is currently not confirmed what initiates brain-associated AD/T3D insulin resistance, and to what extent this contributes to the pathogenesis (De Felice et al., 2014[87]; de la Monte and Wands, 2008[90]; Ferreira et al., 2018[114]; Nguyen et al., 2020[219]; Peng et al., 2024[240]). Before AD can be confirmed as T3D, the associated brain insulin resistance/impaired insulin signalling needs to be confirmed mechanistically to at least partly drive the pathogenesis in patients.

It is unknown/not well studied if AD patients may have preclinical T1D and/or T2D pathology. Given the high prevalence of diabetes and glucose intolerance observed in AD (Barbagallo and Dominguez, 2014[28]), it is reasonable to assume that the remainder of the patients exhibiting glucose tolerance may have asymptomatic preclinical diabetic pathology, which would further strengthen the link between the pathogenesis of these two diseases if this were found to be the case. The association between AD and T2D is well established and widely accepted (Barbagallo and Dominguez, 2014[28]; Peng et al., 2024[240]). However, the overlapping pathological features between T1D and T2D (Beery et al., 2019[33]; Brooks-Worrell et al., 2022[48]; Donga et al., 2013[99]; Guo et al., 2021[133]; Reed et al., 2021[262]), which suggests a diminishing distinction between them, discussed earlier, warrants research to consider how T1D diabetic pathology may encourage AD pathogenesis and improve its diagnosis/prediction (for example, by screening for islet autoantibodies in AD patients or at-risk subjects). Strikingly, an estimated 40 % of people with T1D aged over 30 years at diagnosis are misdiagnosed as T2D (Holt et al., 2021[150]). This raises the question as to how many AD patients diagnosed with T2D may have T1D or LADA. Additionally, T2D manifestation after AD diagnosis could be a misdiagnosis, and subjects could have T1D or LADA. Further research into understanding the shared pathological features between T1D, T2D and AD may strengthen the argument that AD should be reclassified as T3D, and that all these diseases, to at least some degree, are induced by similar pathological mechanisms. Table 1(Tab. 1) (References in Table 1: Addissouky et al., 2024[4]; Beery et al., 2019[33]; Bielka et al., 2024[38]; Brooks-Worrell et al., 2013[49]; Guo et al., 2021[133]; Lomeli et al., 2025[192]; Vankova et al., 2021[320]; Nguyen et al., 2020[219]; Peng et al., 2024[240]; Ravikumar et al., 2023[259]; Reed et al., 2021[262]; Singh et al., 2025[288]) highlights significant overlapping pathological features found in T1D, T2D and AD.

Given that diabetes also raises the risk of subjects developing vascular dementia due to diabetic cardiovascular pathology manifesting as a common post-diagnosis complication, it is warranted to consider how this pathology could contribute to the AD disease phenotype (Cholerton et al., 2016[68]; Elsaka, 2024[111]; Reed et al., 2021[262]). Given that vascular dementia and AD share symptoms such as memory loss and confusion, it is often not easy to distinguish between them in patients (Ng et al., 2025[218]; Semerano et al., 2024[279]). It is currently thought to be uncommon for dementia patients to have 'pure AD' or 'pure vascular dementia', as many patients exhibit pathological hallmarks that are typically associated with both forms (Semerano et al., 2024[279]). Therefore, future research should examine how diabetic pathology can encourage the manifestation of pathology associated with AD and vascular dementia in tandem to induce the dementia phenotype.

## Future Perspectives

T2D, T1D and AD are major global healthcare burdens and their incidence and prevalence continue to rise, meaning that these diseases are major global concerns (Bell and Lain, 2025[34]; Reed et al., 2021[262]; Safiri et al., 2024[271]). Despite the treatment strategies that have been developed, post-diagnosis complications remain prevalent (Kamatham et al., 2024[171]; Leal et al., 2009[181]; Reed et al., 2021[262]; Safiri et al., 2024[271]). Hence, new, more effective therapeutic strategies for these diseases are highly desirable. Additionally, improving detection of these diseases during their preclinical phases is desirable as earlier detection, treatment and intervention strategies are associated with improved prognosis (Herman et al., 2015[146]; Insel et al., 2015[155]; Porsteinsson et al., 2021[250]; Shah et al., 2025[280]). Further, understanding why some individuals remain in the preclinical phases of these diseases for longer than others will also likely generate new therapeutic options to delay disease manifestation or even reverse the disease phenotype.

Glucagon-like peptide-1 (GLP-1) receptor agonists (RAs), which have a much greater half-life than native GLP-1, are currently used to treat T2D and obesity, and mediate their actions through the GLP-1 receptor (GLP-1R) (Drucker, 2024[101]; Reed et al., 2020[261], 2021[262], 2024[263]). Studies have found that, in addition to alleviating hyperglycaemia by augmenting the incretin effect and reducing glucagon secretion, and promoting weight loss via induction of satiety, these analogues have multiple beneficial physiological effects (Perkovic et al., 2024[242]; Reed et al., 2024[263]; Sreenivasan et al., 2024[292]). GLP-1RAs have been shown in several clinical studies, including LEADER, REWIND, and SELECT, to reduce major adverse cardiovascular events in people with T2D and overweight subjects, which has high clinical relevance given the prevalence of cardiovascular disease in diabetes and obesity (Reed et al., 2021[262], 2024[263]; Sawami et al., 2024[278]). Studies have also found that these analogues reduce kidney outcomes in T2D (Sawami et al., 2024[278]). The results of the recent FLOW study found that semaglutide treatment reduced the risk of kidney outcomes and death from cardiovascular causes in people with T2D and chronic kidney disease (Perkovic et al., 2024[242]). Recently, the SOUL trial also found that semaglutide reduced the risk of major adverse cardiovascular events in T2D subjects, but made no difference to kidney events (McGuire et al., 2025[207]).

Interestingly, studies have also found that these analogues reduce insulin requirement and improve glycaemic control in T1D subjects, as well as mitigating weight gain in response to insulin treatment (Edwards et al., 2022[107]; Panjawatanan and Comi, 2025[236]). However, these studies did not investigate whether endogenous insulin levels were increased in response to treatment and whether glucagon levels were reduced. This would be an important area of future research given GLP-1RAs' ability to suppress glucagon secretion, and evidence from studies has shown that GLP-1RAs encourage islet beta-cell survival and proliferation in experimental settings, whilst reducing apoptosis (Reed et al., 2024[263]). Additionally, studies have reported that GLP-1RAs mitigate anti-inflammatory actions, so this may dampen the autoimmunity in T1D and assist any residual islet beta-cell survival and function (Bendotti et al., 2022[35]; Reed et al., 2024[263]). Interestingly, one study found that intraperitoneal injections of GLP-1 and gastrin restored normoglycemia in diabetic NOD mice, a mouse model for T1D, by increasing islet beta-cell mass and insulin content, whilst reducing apoptosis in these cells and dampening the autoimmunity (Suarez-Pinzon et al., 2008[299]). Hence, GLP-1RAs show promise for T1D treatment in the future.

GLP-1RAs also have potential for AD treatment, as evidenced by several animal studies (Reed et al., 2024[263]). Lateral ventricular administration of GLP-1 or exendin-4 in mice reduced endogenous levels of Aß, and hippocampal neuronal cell death due to Aß was prevented in rats by infusion of GLP-1 or exendin-4 (Perry et al., 2003[243]). GLP-1RAs (such as exendin-4 and liraglutide) decreased amyloid precursor protein expression and processing in the brains of AD model mice via GLP-1R activation, and decreased Aß production and plaque formation (McClean et al., 2015[205]; Wang et al., 2016[326]). Liraglutide also reduced the number of Aß plaques in the cortex by an estimated 50 % and soluble amyloid oligomer levels were reduced by 25 % (McClean et al., 2011[206]). Liragultide has also been shown to attenuate insulin resistance induced by insulin overstimulation in human SH-SY5Y cells, resulting in reduced Aß formation and tau hyperphosphorylation (Jantrapirom et al., 2020[164]). Dulaglutide and liraglutide, via GLP-1R signalling, improved cognitive dysfunction in diabetic and AD mice by inhibiting tau protein hyperphosphorylation and NFT formation (Shu et al., 2019[287]; Zhou et al., 2019[350]). In addition to alleviating the Aß and tau hyperphosphorylation pathology associated with AD, GLP-1R activation has also been shown to modulate neuroinflammation and mitochondrial dysfunction, which are also associated with the pathogenesis of this disease (Li et al., 2025[185]). Exendin-4 treatment reduced microglial activation and proinflammatory cytokine production (Qian et al., 2022[255]). GLP-1R activation was also shown to reduce ROS production and protect synaptic function and structure, and GLP-1R deletion impaired mitochondrial function in astrocytes (Li et al., 2025[185]). Thus, animal and *in vitro *studies have shown mechanistically how GLP-1RAs can alleviate AD pathology and they therefore may have the therapeutic potential to provide more effective treatment for this disease in humans (Li et al., 2025[185]; Reed et al., 2024[263]). Recently, one study found that people with T2D treated with GLP-1RAs had a reduced incidence of dementia compared to placebo, which clearly demonstrates the therapeutic potential of these agonists in dementia treatment (Nørgaard et al., 2022[222]). Another study also reported that liraglutide therapy increased cerebral glucose metabolism in AD patients (Reed et al., 2020[261]). Recently, two large-scale phase 3 clinical trials, EVOKE and EVOKE+, investigated the potential of semaglutide treatment to influence AD progression and alter disease-associated biomarkers (Cummings et al., 2025[82]; Li et al., 2025[185]). Although these trials did not confirm the superiority of semaglutide over placebo regarding reducing the rate of AD progression, improvement of disease-related biomarkers was reported. Interestingly, a recent study found that plasma GLP-1 levels were decreased in AD patients and that treatment of SH-SY5Y cells with GLP-1 reduced the generation of Aβ in a dose-dependent manner (Liu et al., 2024[189]); these findings suggest that decreased GLP-1 activity may contribute to AD pathogenesis/progression. The results from the recent EVOKE and EVOKE+ trials suggest that current GLP-1RA-based therapies used in T2D and obesity treatment have negligible therapeutic potential for alleviation of the AD phenotype and will not delay disease progression in patients (Cummings et al., 2026[83]). Future research should therefore aim to elucidate whether GLP-1RAs used at an earlier stage in AD disease can prolong the preclinical phase of AD in at-risk individuals.

Interestingly, one recent study found that human and mouse islets contained bioactive GLP-1, which correlated positively with insulin secretion (Cui et al., 2025[80]). The insulinotropic actions of islet alpha-cell-produced glucagon are well known, but it has been debated/unclear if these cells also augment islet beta-cell insulin secretion through the production of any physiologically relevant levels of GLP-1 (Cui et al., 2025[80]; Reed et al., 2024[263]). Although studies have found that isolated human islet alpha-cells can produce and secrete GLP-1, it remains unclear if this also occurs during *in vivo* scenarios (Campbell et al., 2021[57]). Alpha-TC1/6 cells were shown to secrete GLP-1 under hyperglycemic, hyperlipidemic, or inflammatory conditions in *in vitro *settings (Sancho et al., 2017[275]), suggesting that this hormone may act in a paracrine manner under pathological conditions. Liraglutide was shown to increase alpha-cell GLP-1 expression in human islets (Saikia et al., 2021[272]), in an islet beta-cell GLP-1R-dependent manner, which suggests that islet beta-cell signalling/behaviour can additionally influence GLP-1 production in islet alpha-cells. The recent observation that GLP-1 produced by human islet alpha-cells augments insulin secretion during *in vitro *settings (Cui et al., 2025[80]) warrants further research to determine if this also occurs during *in vivo *settings. In contrast to GLP-1 released by the intestinal L-cells, which has a short half-life due to inactivation by dipeptidyl peptidase-IV enzymes in the circulation (Reed et al., 2020[261], 2021[262], 2024[263]), it is reasonable to assume that the majority of any GLP-1 released from islet alpha-cells would be able to reach GLP-1R on target islet beta-cells before any significant degradation had occurred. Thus, seemingly making any islet alpha-cell GLP-1 secretion more efficient from a bioenergetics point of view. Understanding how islet alpha-cell GLP-1 secretion and subsequent signalling in islet beta-cells may alter between healthy and pathological scenarios in humans could have clinical importance.

Pancreatic fibrosis (PF) is a well-established key pathological feature in chronic pancreatitis, pancreatic ductal adenocarcinoma and cystic fibrosis, which are all associated with a high incidence of pancreatogenic diabetes (Kattner et al., 2026[173]). Interestingly, PF has also been detected in T2D and T1D subjects, and deceased T2D individuals who had > 40 % of the pancreatic tissue area affected by fibrosis, with no evidence of chronic pancreatitis, had been dependent on insulin treatment (Dyson et al., 2025[105]; Kattner et al., 2026[173]; Wright et al., 2023[336]). A recent review suggested that pathological profibrotic signalling between macrophages, pancreatic stellate cells and pancreatic islets, which promotes PF, contributes to islet beta-cell dysfunction/pathology in various forms of diabetes (Kattner et al., 2026[173]). Impaired/pathological crosstalk between the pancreas and the brain due to PF formation/deposition could also have implications in the promotion of pathology associated with AD, given the evidence suggesting pancreatic exocrine function mediates both pathological and beneficial effects on the brain (Bortoletto and Parchem, 2023[43]; Raimundo et al., 2020[257]; Yazawa et al., 2025[338]). The potential of antifibrotic agents for diabetes and AD treatment, therefore, warrants future investigation.

## Conclusions

T2D, T1D and AD are heterogeneous, multifactorial and incompletely understood diseases (Addissouky et al., 2024[4]; Jia et al., 2024[167]; Kamatham et al., 2024[171]; Reed et al., 2021[262]; Virtanen and Knip, 2003[323]; Zhang et al., 2024[344]). The highly heterogeneous nature of these conditions suggests that the pathological biological mechanisms that provoke disease manifestation vary in severity, duration and onset between subjects (Addissouky et al., 2024[4]; Jia and Yu, 2024[168]; Kamatham et al., 2024[171]; Reed et al., 2021[262]; Zhang et al., 2024[344]). Further investigation into the reasons for the heterogeneity within these diseases, and identifying currently unknown environmental risk factors and risk genes that provoke their pathogenesis, will likely yield new and more effective therapeutic options in the future (Addissouky et al., 2024[4]; Giwa et al., 2020[130]; Kamatham et al., 2024[171]; Reed et al., 2021[262], 2024[263]; Safiri et al., 2024[271]; Zajec and Trebušak Podkrajšek, 2022[341]). It is well established that impaired insulin secretion and action are central to the disease phenotypes of both T1D and T2D, but it is still unclear if this is also the case with AD, and this needs to be confirmed before it can be regarded as T3D (Brooks-Worrell et al., 2013[49]; Chaudhary et al., 2025[63]; Nguyen et al., 2020[219]; Peng et al., 2024[240]; Ravikumar et al., 2023[259]; Reed et al., 2021[262]). Evidence suggests that GLP-1RAs can impact the phenotypes of all these diseases (Li et al., 2025[185]; Panjawatanan and Comi, 2025[236]; Reed et al., 2020[261], 2021[262], 2024[263]), and will likely provide more effective therapeutic strategies in the future. Historically, these diseases were viewed as distinct, but given the emerging evidence that they share similar pathological features, and as AD and DM are risk factors for each other (Beery et al., 2019[33]; Brooks-Worrell et al., 2022[48]; Donga et al., 2013[99]; Guo et al., 2021[133]; Jeong et al., 2025[166]; Li et al., 2025[184]; Nguyen et al., 2020[219]; Peng et al., 2024[240]; Reed et al., 2021[262]), this view is now being questioned. Future research should aim to understand these features and bidirectional relationships mechanistically, which may yield new therapeutic strategies to alleviate the disease phenotypes.

## Declaration

### Acknowledgements

JR was a recipient of the Knowledge Economy Skills Scholarship (KESS) II UK PhD studentship. We thank the members of the VK laboratory.

### Conflict of interest

The authors declare that they have no conflict of interest.

### Artificial Intelligence (AI) - assisted technology

Artificial intelligence was not used in the preparation of this manuscript.

### Authors' Contributions

JR conceptualised and produced the draft manuscript and figures. SCB and VK contributed equally to reviewing and editing the draft manuscript. All authors have reviewed and approved the final version of the manuscript.

## Figures and Tables

**Table 1:**
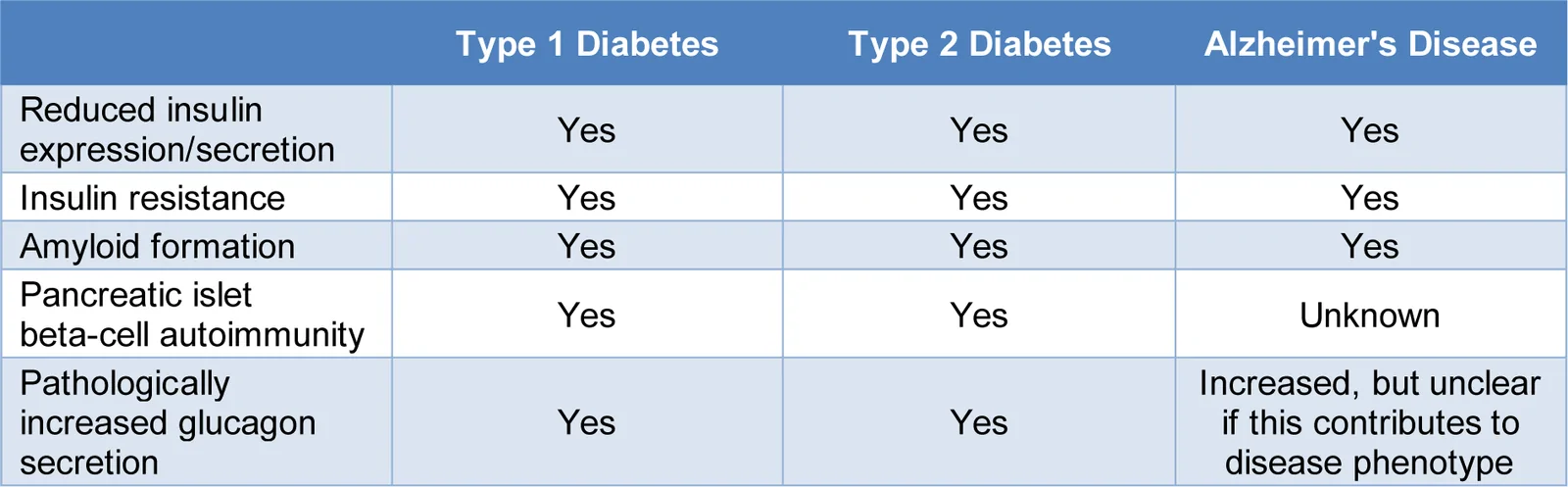
Significant overlapping pathological features demonstrated in studies between a subset or greater of T1D, T2D and AD patients. This table was generated using information available from (Addissouky et al., 2024; Beery et al., 2019; Bielka et al., 2024; Brooks-Worrell et al., 2013; Guo et al., 2021; Lomeli et al., 2025; Vankova et al., 2021; Nguyen et al., 2020; Peng et al., 2024; Ravikumar et al., 2023; Reed et al., 2021; Singh et al., 2025).

**Figure 1:**
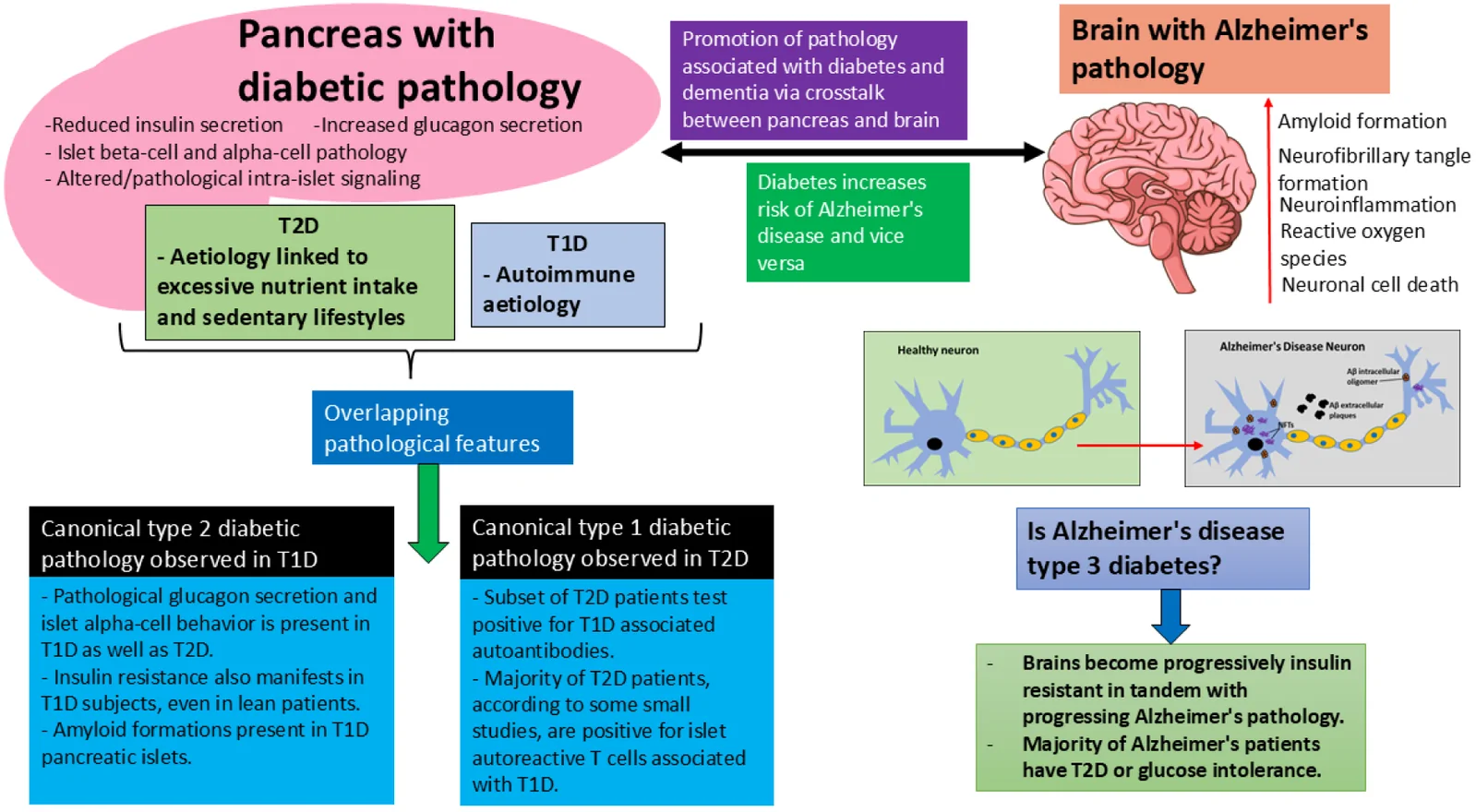
Graphical abstract
